# ORFans in Mitochondrial Genomes of Marine Polychaete *Polydora*

**DOI:** 10.1093/gbe/evad219

**Published:** 2023-11-29

**Authors:** Maria Selifanova, Oleg Demianchenko, Elizaveta Noskova, Egor Pitikov, Denis Skvortsov, Jana Drozd, Nika Vatolkina, Polina Apel, Ekaterina Kolodyazhnaya, Margarita A Ezhova, Alexander B Tzetlin, Tatiana V Neretina, Dmitry A Knorre

**Affiliations:** Faculty of Bioengineering and Bioinformatics, Lomonosov Moscow State University, Moscow, Russia; Faculty of Bioengineering and Bioinformatics, Lomonosov Moscow State University, Moscow, Russia; Faculty of Bioengineering and Bioinformatics, Lomonosov Moscow State University, Moscow, Russia; Faculty of Bioengineering and Bioinformatics, Lomonosov Moscow State University, Moscow, Russia; Faculty of Bioengineering and Bioinformatics, Lomonosov Moscow State University, Moscow, Russia; Faculty of Bioengineering and Bioinformatics, Lomonosov Moscow State University, Moscow, Russia; Faculty of Bioengineering and Bioinformatics, Lomonosov Moscow State University, Moscow, Russia; Faculty of Bioengineering and Bioinformatics, Lomonosov Moscow State University, Moscow, Russia; Faculty of Bioengineering and Bioinformatics, Lomonosov Moscow State University, Moscow, Russia; Pertsov White Sea Biological Station, Faculty of Biology, Lomonosov Moscow State University, Moscow, Russia; Center of Life Sciences, Skolkovo Institute of Science and Technology, Moscow, Russia; Pertsov White Sea Biological Station, Faculty of Biology, Lomonosov Moscow State University, Moscow, Russia; Faculty of Bioengineering and Bioinformatics, Lomonosov Moscow State University, Moscow, Russia; Pertsov White Sea Biological Station, Faculty of Biology, Lomonosov Moscow State University, Moscow, Russia; Institute for Information Transmission Problems (Kharkevich Institute), Russian Academy of Science, Moscow, Russia; Pertsov White Sea Biological Station, Faculty of Biology, Lomonosov Moscow State University, Moscow, Russia; Belozersky Institute of Physico-Chemical Biology, Lomonosov Moscow State University, Moscow, Russia

**Keywords:** mitochondrial DNA, pseudogenes, invertebrates, gene duplication, neofunctionalization

## Abstract

Most characterized metazoan mitochondrial genomes are compact and encode a small set of proteins that are essential for oxidative phosphorylation, as well as rRNA and tRNA for their expression. However, in rare cases, invertebrate taxa have additional open reading frames (ORFs) in their mtDNA sequences. Here, we sequenced and analyzed the mitochondrial genome of a polychaete worm, *Polydora cf. ciliata*, part of whose life cycle takes place in low-oxygen conditions. In the mitogenome, we found three “ORFan” regions (544, 1,060, and 427 bp) that have no resemblance to any standard metazoan mtDNA gene but lack stop codons in one of the reading frames. Similar regions are found in the mitochondrial genomes of three other *Polydora* species and *Bocardiella hamata*. All five species share the same gene order in their mitogenomes, which differ from that of other known Spionidae mitogenomes. By analyzing the ORFan sequences, we found that they are under purifying selection pressure and contain conservative regions. The codon adaptation indices (CAIs) of the ORFan genes were in the same range of values as the CAI of conventional protein-coding genes in corresponding mitochondrial genomes. The analysis of the *P. cf. ciliata* mitochondrial transcriptome showed that ORFan-544, ORFan-427, and a portion of the ORFan-1060 are transcribed. Together, this suggests that ORFan-544 and ORFan-427 encode functional proteins. It is likely that the ORFans originated when the *Polydora/Bocardiella* species complex separated from the rest of the Spionidae, and this event coincided with massive gene rearrangements in their mitochondrial genomes and tRNA-Met duplication.

**Significance**

Metazoan mitochondrial genomes usually contain a conservative set of genes and features. However, mitogenomes of some species contain ORFans—putative protein-coding genes (PCGs) without clear homology with other known sequences. In this study, we analyzed three ORFans in the mitochondria of the marine annelid worms of the genera *Polydora* and *Bocardiella*. Sequence analysis of the ORFans suggests they contain conservative regions and are likely translated into functional proteins. Our study features an uncommon case where new PCGs emerged in the mitochondrial genomes of metazoa.

## Introduction

Mitochondrial genomes of Metazoa are characterized by extraordinary diversity of their genomic features in some taxa and consistency in others. A typical mitogenome of bilaterian species usually contains 37 genes encoding 13 proteins, 2 ribosomal RNA genes (rRNAs), and 22 transfer RNA genes (tRNAs), as well as a single noncoding “control region” (CR) with regulatory signals for replication and transcription. Such features also include conserved genetic architecture and compact gene organization, which implies the absence of additional noncoding regions, NCRs (Ghiselli et al. 2021). At the same time, nonbilaterians and some bilaterian taxa show high levels of diversity in mitogenome gene composition and chromosome structure and have reassignments in genetic codes. Several species also have multipartite genomes and have lost some of the tRNA genes (Lavrov and Pett 2016).

Moreover, some invertebrate species have additional NCRs and “unconventional” genes in their mitochondrial genomes (Milani et al. 2013). For instance, the mitochondrial genomes of demosponges and some glass sponges harbor an additional ATP-synthase gene, *ATP9* (Lavrov et al. 2005; Haen et al. 2007); octocoral mitogenomes contain a DNA-repair mutS gene (Pont-Kingdon et al. 1995). Unusual regions were found in mollusk mitochondrial genomes. Some of the bivalves have doubly uniparental mtDNA inheritance (DUI), where one of the mtDNA variants is inherited strictly paternally and the other strictly maternally (Zouros et al. 1994; Breton et al. 2007). Mitochondrial genomes of such bivalves usually contain additional open reading frames (ORFs) linked to sex—these ORFs with no detectable homology and unknown function are referred to as ORFans (Breton et al. 2009; Milani et al. 2013; Mitchell et al. 2016). There is evidence which supports these genes playing a role in mitochondrial DNA inheritance (Mitchell et al. 2016). Additionally, in male-transmitted mtDNA mitochondrial genomes of bivalves, some standard PCGs are significantly elongated, whereas additional sequences show no homology to any other known sequence (Ghiselli et al. 2021). As a result, the size of the corresponding protein is significantly increased (Tassé et al. 2022).

Mitogenomes of Cnidaria, Porifera, and Ctenophora species also contain ORFans (Flot and Tillier 2007; Haen et al. 2007; Breton et al. 2009; Schultz et al. 2020). Mitochondrial genomes of Platyctenida, the benthic Ctenophores, encode ORFans containing predicted transmembrane domains (Arafat et al. 2018). Furthermore, some of the ORFans have been shown to have a conserved structure across related species (Guerra et al. 2019; Schultz et al. 2020). However, in most cases, the functions and origin of the ORFans remain uncertain.

Spionids, which make up one of the most diverse families of marine annelids, are characterized by the amazing plasticity of their lifestyles and life cycles. Spionids occur in a wide variety of habitats from the intertidal zone to the deep sea, sometimes forming dense benthic assemblages (Blake et al. 2020). Their long-living larvae can be the dominant forms of larval plankton in the pelagic zone. Individuals may extend their palps from burrows or tubes to filter particles from the water; in other cases, the worms are surface deposit feeders. In general, Spionids are characterized by life cycle adaptations such as a wide variety of sperm types and types of fertilization, asexual reproduction (architomy and paratomy), poecilogony, and in some cases, adelphophagy (Radashevsky and Migotto 2017; Sato-Okoshi et al. 2017). Spionids of genera close to the genus *Polydora* are characterized by an unusual ability to drill hard calcareous substrates. In most cases, they drill into mollusk shells, often being pests of commercially cultivated mollusks (e.g., oysters). At the same time, these animals are often able to build tubes and live in sediment (Blake et al. 2020).

The architecture of the annelid mitochondrial genome is generally well conserved (Weigert et al. 2016), with some notable exceptions, for example, *Ramisyllis multicaudata* from the family Syllide (Aguado et al. 2015). Moreover, recently three complete mitogenomes of shell-boring *Polydora* species (Spionidae family), *Polydora websteri*, *Polydora brevipalpa*, and *Polydora hoplura*, were sequenced (Ye et al. 2021; Lee and Lee 2022). Analysis of two related species, *P. websteri* and *P. brevipalpa*, and comparisons with other sedentary animals showed that *Polydora* mitogenomes have some unusual features: the gene order has been changed, and there are four NCRs that are longer than 500 base pairs (bp). Analysis of four large NCRs in both genomes has shown that none of them exhibited sequence similarity to the nearby coding regions. At the same time, several ORFs > 50 amino acid (a.a.) residues were detected in NCRs, but BlastP analysis found no hits with known proteins for any of the putative protein products (Ye et al. 2021). The mitogenome of *P. hoplura* has been shown to have similar architecture, including the presence of NCRs (Lee and Lee 2022).

In this study, we sequenced and assembled the mitogenome of an additional *Polydora* specimen collected in Biofiltry Bay near White Sea Biological Station, *Polydora cf. ciliata* (Radashevsky and Pankova 2006, 2013). In different parts of this small sea, *P. ciliata* bores into the shells of littoral gastropods, such as *Littorina littorea* on the Solovetsky Islands, or lives in a very rich organic sediment on the border of sulfur-donor (low-oxygen conditions) zones in small shallow inlets (Kandalaksha Bay).

We analyzed the mitogenomes of this and previously described *Polydora* and other Spionida species and found that three out of four NCR regions lack stop codons along the entire length of the region in one of the reading frames. Moreover, these regions accumulated more synonymous than nonsynonymous mutations. We also found that two of these regions and two portions of the third region are transcribed. This implies that these regions might encode functional proteins.

## Results

**Fig. 1.**
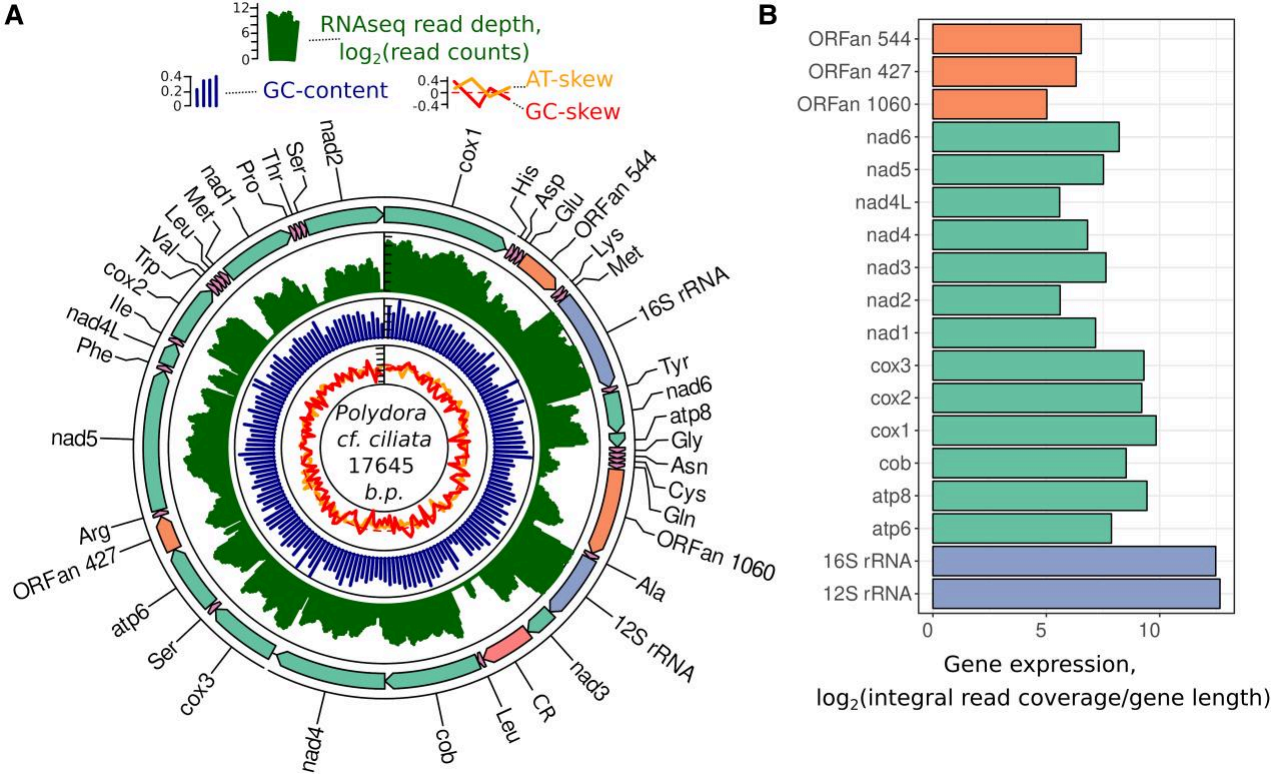
Mitochondrial DNA of *P. cf. ciliata* comprises three ORFan regions. (*A*) mtDNA sequence map. The outer track represents boundaries of standard mitochondrial PCGs, CR, ORFans, tRNAs, and rRNAs. Arrow direction corresponds to the coding strand. Three-letter a.a. codes denote the corresponding tRNAs. Next (green) track represents RNAseq sequence results as cDNA read depth; it is followed by sequence statistics of GC content and nucleotide skews. (*B*) Relative expression levels of rRNA, PGCs, and ORFan regions.

We collected a specimen of *P. cf ciliata* in the Biofiltry Bay, a small shallow water inlet in the Kandalaksha Bay of the White Sea (see Materials and Methods for details). Isolating and sequencing the total genomic DNA from this specimen enabled the assembly of the mitochondrial genome. In this genome, we found all standard metazoan protein-coding genes (PCGs), rRNAs, tRNAs, duplicated tRNAs, and four additional regions that were also previously found in *P. websteri*, *P. brevipalpa*, and *P. hoplura* species (Ye et al. 2021; Lee and Lee 2022). Similar to other polychaetes, all genes were encoded on a single strand (fig. 1*A*). We isolated RNA and produced cDNA from another *P. cf. ciliata* specimen using reverse-transcriptase reaction with random primers. The cDNA was sequenced using the Illumina platform (see Materials and Methods for details). Figure 1*A* shows the high coverage of PCGs and rRNAs, punctuated by low-coverage gaps between them. Two regions, 427 and 544 bp in length, exhibited high cDNA coverage, mirroring the expression levels of other PCGs within the *P. cf. ciliata* mitochondrial genome (fig. 1*B*). Concurrently, the 1,060 bp region encoded two separated RNA transcripts. Finally, the fourth region, located downstream of the *nad3* gene, contained only a minor segment with RNAseq read mapping (fig. 1*A*, supplementary fig. S1, Supplementary Material online).

**Fig. 2.**
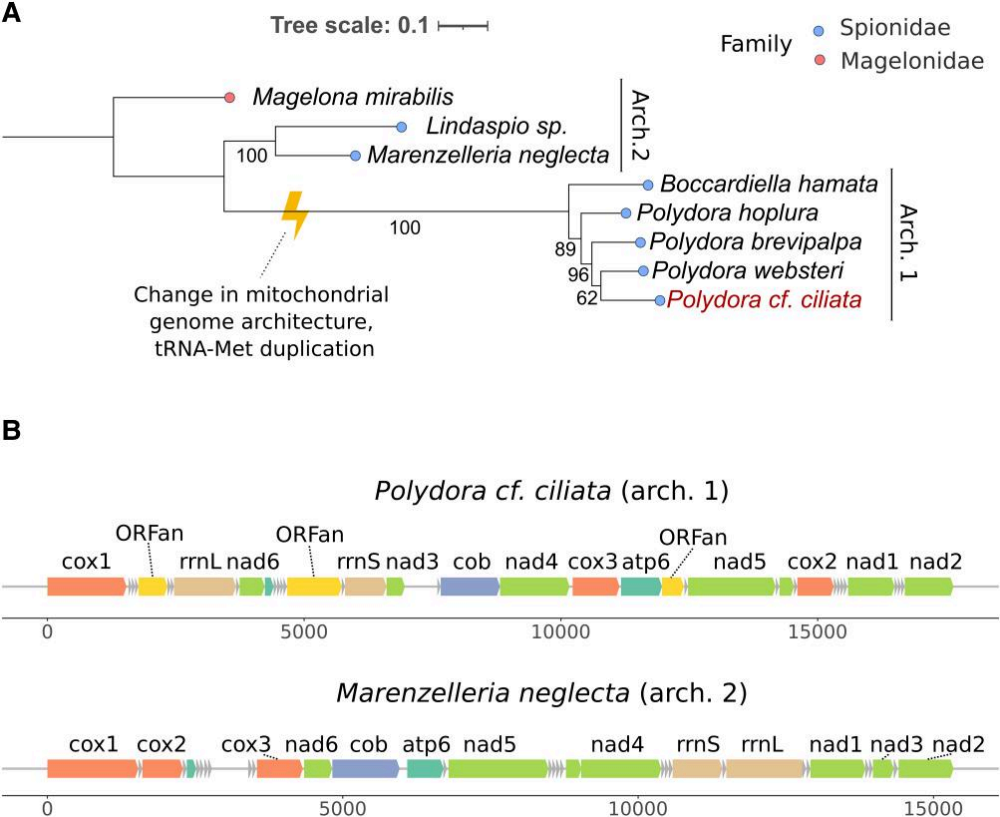
Species tree and mitochondrial genome architecture of the Spionidae. (*A*) Phylogenetic tree of 7 Spionid mitogenomes based on coding sequences of 12 standard PCGs, excluding *ATP8* (concatenated codon multiple sequence alignments of PCGs). The tree was constructed using the ML method, and ModelFinder and partition testing with segments each corresponding to a specific gene were used. Numbers represent bootstrap percentages, which were determined by 500 replicates. The tree is rooted with the *M. mirabilis* mitogenome as an outgroup. The mitogenome of *P. cf. ciliata* sequenced and annotated within this project is highlighted. (*B*) Two types of genome architectures characteristic of Spionid species mitogenomes. Arch. 1 is characteristic of mitogenomes before ORFan emergence in lineage evolution (*Lindaspio* sp. *i ZZ-2021*, *M. neglecta*, as well as many other annelids); arch. 2 is unique for available *Polydora* sp. and *B. hamata* mitogenomes.

Phylogenetic analysis of Spionidae and *Polydora* mitochondrial genomes available on GeneBank, with *Pseudopotamilla reniformis* as an outgroup, was done on 12 standard OXPHOS genes (excluding nonconservative *ATP8*). We translated all genes with mitochondrial invertebrate genetic code and aligned the resulting a.a. sequences. We then concatenated alignments and built a phylogenetic tree (fig. 2). The analysis showed that the sequenced mitochondrial genome—*P. cf. ciliata—*is similar to *P. websteri*. Phylogenetic trees built from codon alignments were consistent with those built from a.a. alignments. To clarify *P. cf. ciliata* phylogeny, we then built phylogenetic trees from alignments of 16S, 18S, and 28S Polydora and Spionidae genes. According to the analysis of 16S rRNA sequences, *P. cf. ciliata* is closely related to *P. onagowaensis*, although the construction of phylogenies from individual 16S, 18S, and 28S genes did not allow us to obtain reliable topologies (supplementary figs. S2–S4, Supplementary Material online).

Available *Polydora* mitogenomes form a cluster that also includes the *Bocardiella hamata* mitochondrial genome (fig. 2). mtDNA sequences comprising this cluster harbor four additional regions and have a unique gene order which distinguishes them from the other Spionidae species. To test if the four unconventional regions encode proteins, we translated them in all six reading frames with invertebrate mitochondrial genetic code. We found that three of the regions contained one frame without stop codons; this was true in the cases of all four *Polydora* and *B. hamata* mitogenomes. Supplementary fig. S5, Supplementary Material online shows distributions of stop codons in the mitogenomes in all six frames. In these 3 regions, all but 1 frame contained 4–39 stop codons per frame. The fourth region, located downstream of the *nad3* gene, contained multiple stop codons in all frames. Furthermore, this fourth region had lower GC content than any of the PCGs. Finally, it contained sequences capable of folding into stable secondary structure elements, according to the RNASurface algorithm (see Materials and Methods and supplementary fig. S6, Supplementary Material online). The replication origin predicted by MitoZ (Meng et al. 2019) also falls in this region. Therefore, we annotated it as a CR of the mitochondrial DNA, whereas the other three regions we annotated as ORFans: ORFan-544, ORFan-1060, and ORFan-427 according to their size in nucleotides.

Thereafter, we tried to find sequences with a homology to the three ORFans identified in the *P. cf. ciliata* mitogenome. First, we searched for homology among nucleotide and protein sequences available in the National Center for Biotechnology Information (NCBI) databases, Non-Redundant Protein Database (NRPD), and UniProt Knowledgebase (UniProtKB). Blast-based algorithms found no discernible homology for any of the *P. cf. ciliata* ORFan a.a. and nucleotide sequences, with the exception of ORFan regions in other *Polydora* and *Bocardiella* mitochondrial genomes. In order to detect more remote homologies, we also performed a hidden Markov model (HMM) (see Materials and Methods) for the ORFans in five species. Moreover, tertiary structure predictions along with structure similarity searches for three ORFans in five species were performed using @TOME and I-TASSER (supplementary table S3, Supplementary Material online). However, these approaches did not reveal sequences that showed any resemblance to any of the three ORFans in the studied species. More detailed information on the remote homology search results can be found in the Supplementary material (supplementary table S3 and text S1, Supplementary Material online).

**Fig. 3.**
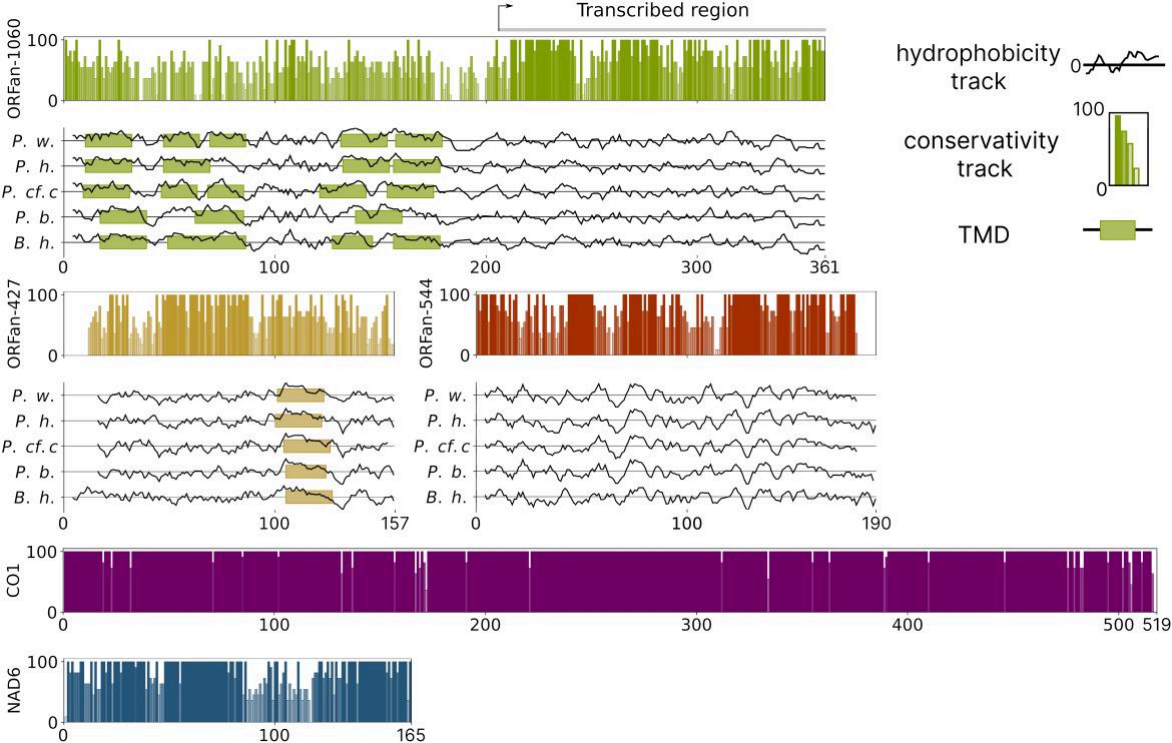
Conservativity score and hydrophobicity of the ORFans. Conservativity was calculated and plotted using gene-specific a.a. alignments across all five ORFan-carrying species. Hydropathy profiles were calculated with the Kyte and Doolitte method via the ProtScale tool at ExPASy; transmembrane domains (TMD) were detected using InterProScan. Prefixes in hydrophobicity and transmembrane tracks denote studied species: *P.w.*, *P. websteri*; *P.h.*, *P. hoplura*; *P. cf. c.*, *P. cf ciliata*; *P.b.*, *P. brevipalpa*; and *B.h.*, *B. hamata.* Hydrophobicity and transmembrane tracks were adjusted to match gene alignments.

Meanwhile, the alignment of the ORFans from the *P. cf. ciliata* mitogenome to the corresponding ORFans in other mitogenomes revealed that all of them have conserved regions (fig. 3). To obtain a conservation score track, we built alignments of the three ORFans as well as two conventional PCGs, *NAD6* and *CO1*, in all five species of consideration. In the ORFans, both regions with high and low conservation scores were found. For ORFan-1060, the high conservation scores were detected in the C-terminus part: 213–240, 261–282 in a.a. annotation—the same regions that exhibited coverage of the RNAseq reads. ORFan-427 also has several regions with high conservation scores: 7–15 and 32–72 regions, a.a. annotation. ORFan-544 had the largest number of regions with high conservation scores: 1–14, 45–56, 71–97, and 119–180 in a.a. annotation.

To determine the possible function and structure of the ORFans, we predicted the domain architectures of ORFans and made hydrophobicity tracks in the five studied species using Phobius integrated into InterProScan (see Materials and Methods). Phobius predicted the presence of several (4 or 5) transmembrane domains in ORFan-1060 in the N-terminus part of the putative protein in all species, although all of them fell into the region which did not produce a stable transcript (fig. 3). Meanwhile, ORFan-427 and its homologs in other studied Polydora species harbored a single transmembrane domain located closer to the C-terminus of the protein in the same position in all analyzed species (fig. 3). Apart from the transmembrane domains in ORFan-427 and ORFan-1060, algorithms integrated into InterProScan, MotifScan, and TPRpred did not reveal any domains and structural motifs in the ORFans.

**Fig. 4.**
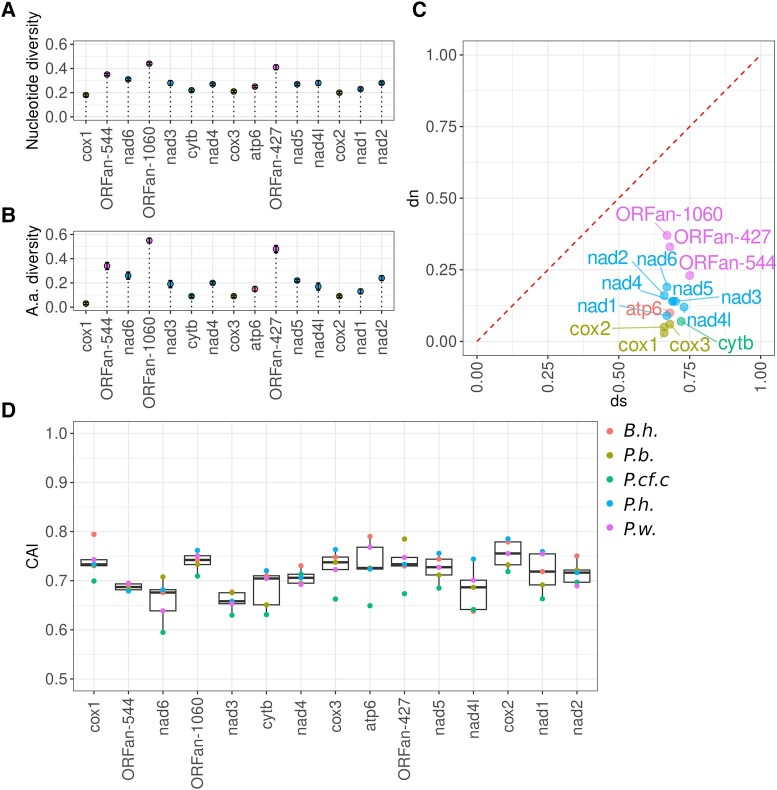
ORFans evolve faster than conventional PCGs but are under the pressure of purifying selection. Nucleotide (*A*) and a.a. (*B*) diversity of the ORFans and standard mitochondrial PCGs calculated as p-distances. Values of nucleotide and a.a. p-distance variance are obtained by the bootstrap method with 500 replicates. (*C*) Proportion of synonymous (ds) and nonsynonymous mutations (dn). The codon-based Z-test for purifying selection shows that we should reject the null hypothesis of neutral evolution (dn = ds) in favor of the alternative hypothesis of purifying selection for all standard OXPHOS genes as well as for all ORFan genes (*P* value < 0.05). (*D*) Codon adaptation index of mitochondrial PCGs and ORFans calculated using Python CAI (Lee 2018).

The absence of stop codons in one of the reading frames suggests that the gene was either recently pseudogenized or encodes a functional protein. In the latter case, one would expect a purifying selection pressure on their protein sequence, which should be expressed in an increased dN/dS ratio. To test this, we calculated nucleotide and a.a. p-distances as well as the dN/dS ratio for PCGs in *Polydora* and *Bocardiella* genomes (fig. 4). ORFan genes appeared to be the most diverse of the analyzed genomes (fig. 4*A* and *B*). Thus, we consider them to be the fastest-evolving PCGs in the mitogenomes. Accordingly, as shown in fig. 4*C*, dN/dS ratios in ORFans are also slightly higher than in other PCGs. However, the codon-based Z-test for purifying selection showed that along with the other PCGs, ORFans are subject to purifying selection in all genomes investigated, which is statistically significant (*P* value < 0.001). This finding indicates that ORFans are translated and are under purifying selection pressure which takes place at the level of a.a. sequences. Furthermore, we calculated codon adaptation indices (CAIs) (see Materials and Methods) for PCG and ORFans, using information from all other genes as a background. CAIs reflect codon usage bias and are expected to be high in weakly expressed genes. Figure 4*D* shows that the CAI of ORFan genes is similar to the CAI of other PCGs; in some worm mitogenomes, it is even higher than the CAIs of some conventional PCGs, for example, *NAD3* and *NAD4L*.

## Discussion

Mitochondria evolved from a proteobacteria endosymbiont (Martijn et al. 2018). After symbiogenesis, its genome underwent a significant reduction. Many genes have been lost or transferred to the nuclear genome, whereas acquisitions of new elements in mtDNA via horizontal transfer were extremely rare (Janouškovec et al. 2017). One of the exceptions is the mutS gene in the mitochondrial genomes of octocorals (Pont-Kingdon et al. 1998). Gene duplications within mitochondrial genomes are also uncommon, although reported in several taxa. For instance, the mitogenome of the nematode *Caenorhabditis briggsae* contains a pseudogenized copy of the *NAD5* gene (Raboin et al. 2010). The mitochondrial genome of *Chaetopterus variopedatus*, a parchment worm, contains a duplicated *COX1* gene and a portion of a duplicated *NAD3* gene. The latter is adjacent to an unannotated region (Weigert et al. 2016).

In this study, we expand these rare examples with the ORFans located in the mitochondrial genomes of Polychaete worms in the genus *Polydora*, which belongs to the Spionidae family. Spionidae is divided into two subfamilies that appear to be monophyletic groups: the first is subfamily Spioninae Söderström, 1920, which includes the tribe Polydorini (including the genera *Polydora* and *Bocardiella*) and the second is subfamily Nerininae Söderström, 1920, which includes the genera *Marenzelleria* and *Lindaspio*. The tree shown in fig. 2 supports the modern view of the system and phylogeny of Spionidae (Blake et al. 2020).

Analysis of the mitochondrial gene expression showed that two of the three ORFans, namely ORFan-427 and ORFan-544, are transcribed and the levels of their transcripts are similar to the other mitochondrial PCGs, such as *NAD4L* and *NAD2*. This makes a striking contrast to the predicted CR, where RNAseq did not reveal stable RNA products (fig. 1). At the same time, sequencing of the ORFan-1060 cDNA revealed two shorter products. This implies that ORFan-1060 yields only shorter peptides or is not translated into a protein at all. Accordingly, fig. 3 shows that only the C-terminal region of this protein shows a high degree of a.a. sequence conservation. Moreover, the RNAseq results are consistent with our observation that the a.a. diversity and dN/dS ratio of ORFan-1060 is higher than corresponding values calculated for the two other ORFans (fig. 4).

Our analysis suggests that two ORFans are transcribed and possibly translated into functional proteins. With the exception of the common mitochondrial PGCs, ORFans are the longest regions in mitochondrial genomes that are devoid of stop codons in one of the reading frames. Supplementary fig. S5, Supplementary Material online features the distribution of stop codons in the mitochondrial genomes of Polydorides in three reading frames of the coding strand. Moreover, some regions of ORFans show a high degree of conservation, and ORFan-427 contains a predicted transmembrane domain in four *Polydora* species in a similar genomic position (fig. 3). Finally, ORFan sequences showed evidence of purifying selection: their dN/dS values are 0.552 (ORFan-1060), 0.485 (ORFan-427), and 0.307 (ORFan-544) (fig. 4*C*). It should be mentioned that these analyses cannot exclude the possibility that ORFans recently became nonfunctional (useless to worm mitochondria) in some of the studied species and did not accumulate enough mutations to detect them. However, the scenario suggesting that ORFans became pseudogenes in all five species is highly unlikely.

The transmembrane domain predicted in ORFan-427 (fig. 3) suggests that this putative protein is incorporated into the mitochondrial inner membrane. Furthermore, given the absence of molecular machinery facilitating protein export from the mitochondria under normal conditions, it is plausible that ORFans are normally retained within the mitochondria. Nevertheless, due to the lack of even remote homology of the ORFans, it is not possible to speculate about their function.

Mitochondrial ORFans can have three possible origins: 1) They were present in the mitochondrial genome of the metazoan's common ancestor mtDNA and preserved in some clades, 2) they arose through duplication of conventional mtDNA genes, or 3) they emerged via horizontal transfer from other genomes, for example, nuclear DNA of the same species or viral DNA. In the case of *Polydora* and *Bocardiella* species, the first explanation is highly unlikely given that all other sequenced annelid mitogenomes are devoid of ORFans sharing homology with Polydora ORFans discussed in this study. However, there is not enough data to discriminate between the second and third possibilities. On the one hand, ORFans have no homology with any other mitochondrial PCG. Moreover, many other annelid species contain type II introns inside mitochondrial PGCs (Vallès et al. 2008; Richter et al. 2015; Bernardino et al. 2017; Kobayashi et al. 2022), which were likely incorporated via horizontal gene transfer from a virus or bacteria (Vallès et al. 2008). On the other hand, if ORFans have recently been transferred into the mitochondrial genome from a viral or any nuclear genome, we would expect significant differences in AT content, AT skew, and GC skew. Figure 1 and supplementary fig. S6, Supplementary Material online show that the genomic parameters of ORFans fall in range of the same parameters of conventional PCGs.

We suggest that ORFans emerged as a result of intrachromosomal rearrangement of the *Polydora* ancestor and then underwent a stage of rapid evolution that destroyed the phylogenetic signal that could allow us to identify their origin. Indeed, tandem duplications in mitochondrial genomes are usually followed by random or nonrandom gene loss. These events result in mitochondrial DNA rearrangements (Lavrov et al. 2002; San Mauro et al. 2006). At the same time, one of the duplicated genes can undergo rapid evolution and neofunctionalization (Conant and Wolfe 2008). Duplication of methionine tRNA in *Polydora* and *Bocardiella* mitogenomes (fig. 2) suggests that the mitochondrial genome or portion of the mtDNA of a common ancestor of *Polydora* and *Bocardiella* worms was duplicated. After that, duplicated genes might be either lost or repurposed in their role and function. The fact that genomic rearrangement and ORFan emergence are on the same branch of the phylogenetic tree (fig. 2) supports this assumption. The presence of transmembrane domains in ORFan-1060 and ORFan-427, which are prevalent in mitochondrial coding genes, and the rarity of HGT to metazoan mitochondria are also in line with the duplication-and-loss scenario of Annelid ORFan origin. However, it should be mentioned that the nuclear genome also encodes ∼20% of proteins with transmembrane domains (Krogh et al. 2001) and there are reported cases of horizontal gene transfer to Annelida mitogenomes (Vallès et al. 2008; Richter et al. 2015; Bernardino et al. 2017; Kobayashi et al. 2022).

Although the mitochondrial genome architecture of annelids is conserved, there are some notable exceptions. For example, the mitochondrial genome was shuffled during evolution of another Annelida family, Syllidae (Aguado et al. 2016). Moreover, Syllidae mitogenomes contain NCRs up to 445 bp long (Aguado et al. 2016). Some of these NCRs are devoid of stop codons and are thus similar to the ORFans described in this work. However, with the exception of Polydora/Bocardiella species, there are currently not enough complete mitochondrial genomes to perform a comparative analysis of these regions. In addition, as we noted above, type II introns were found in the annelid mitochondrial genomes. The presence of unusual features in mitochondrial genomes of distant Annelidae representatives suggests that they have some prerequisites that lead to the destabilization of the mitochondrial genome architecture and emergence of uncommon features in some branches of the taxon.

To summarize, in this study, we analyzed three putative PCGs in mitochondrial genomes of several polychaete species belonging to the genus *Polydora* and *Bocardiella*. Two of these genes are transcribed and likely to encode proteins with conservative a.a. sequences, although we detected no homologs of the ORFans outside of this group. Moreover, their possible functional role cannot be deduced from their sequences, although it might be noted that all species with ORFans in their mitogenomes rely on drilling hard calcareous substrates. ORFans in *Polydora* and *Bocardiella* species provide a very rare example where new genes emerged in the mitochondrial genome and likely acquired a new function.

## Materials and Methods

### Specimen Collection and Identification

Live specimens of *P. cf. ciliata* (Johnston 1838) were collected with the help of SCUBA divers on the soft bottom in the Biofiltry Bay, a small shallow water inlet in the Kandalaksha Bay of the White Sea, at a depth of 7–8 m (66°32ʹ21ʹʹN, 33°09ʹ59ʹʹE), in close vicinity to the White Sea Biological Station of the MSU.

Spionid polychaetes *P. ciliata* were first described by Johnston in 1838 (Johnston 1838) as inhabitants of muddy tubes in shallow waters (littoral) on the coast of Scotland (Berwick Bay). Since then, worms very similar in morphology to those described by Johnston have been found in both the North Atlantic and the North Pacific. Representatives of the species are described as mass inhabitants of soft bottom substrates, as living in silty pipes, or as worms boring holes in mollusk shells (see (Mustaquim 1986; Radashevsky and Pankova 2006) for review). It was first recorded in the White Sea about 65 years ago (Sveshnikov 1958). At different times, the inhabitants of silty tubes and bored shells were described as separate but hardly recognizable morphological species (Annenkova 1934); apparently, there is a group of very close morphological species here. However, despite many years of efforts (Kendall 1980; Mustaquim 1986; Manchenko and Radashevsky 1993; Radashevsky and Pankova 2006), this issue still remains unresolved, and it seems to us correct to define our worms as *P. cf. ciliata* (Johnston 1838).

### DNA Isolation and Next-Generation Sequencing

DNA was extracted using a Diatom DNA kit (Isogen) according to the manufacturer’s recommendations. DNA libraries were constructed using the NEBNext Ultra II DNA Library Prep Kit by New England Biolabs (NEB) and the NEBNext Multiplex Oligos for Illumina (Index Primers Set 1) by NEB following the manufacturer's protocol. The samples were amplified using ten cycles of polymerase chain reaction (PCR). The constructed libraries were sequenced on an Illumina MiniSeq with a paired-end read length of 150.

### Assembly and Annotation

The quality of the library was assessed using the fastqc program (Andrews 2010). Primary processing of readings (trimming) was carried out using the trimmomatic program (Bolger et al. 2014). The final assembly was made using SPAdes spades-3.14.0 (Antipov et al. 2019), and the longest contig containing several mitochondrial genes was defined as mitochondrial. The assembly was then refined using Sanger sequencing of unannotated regions and the *COX3* gene, as well as Illumina sequencing of mitocontig parts obtained using long-range PCR (the primers are listed in supplementary tables S1 and S2, Supplementary Material online).

Annotation of the genome was retrieved using MitoZ (Meng et al. 2019). We manually specified the coordinates of PGCs by aligning the genes with the corresponding genes of other annelid species. The CR was identified using the RNASurface algorithm (Soldatov et al. 2014) which reflects the significance of putative secondary structure in the region, as well as by examining GC content distribution along the genome and locating the replication origin using MitoZ (supplementary fig. S5, Supplementary Material online). The *ATP8* gene was identified in all five considered mitogenomes using multiple alignments of *ATP8* genes from related species with a potential *ATP8* ORF (supplementary fig. S6, Supplementary Material online).

In order to find ORFans in all genomes considered, we first divided genomes into codons in all reading frames and identified invertebrate mitochondrial stop codons. Then, pairwise distances between neighboring stop codons were calculated and the resulting genomic regions were filtered by length (360 bp). Long regions were divided by tRNA genes boundaries (if intersected). The resulting possible PCGs were filtered by length again (210 bp). All steps were performed using R programming language (dplyr и Biostrings libraries). Codon alignments were used to establish exact ORFan (PCGs in unannotated regions) boundaries.

A program code for constructing a schematic image of the mentioned maps and annotations was written in R using circlize library (Gu et al. 2014) and tidyverse libraries (Wickham et al. 2019). Sequence statistics for the plot were computed with Biostrings (Pagès et al. 2023) and dplyr (Wickham et al. 2022). The mapping was performed using bowtie2 (Langmead and Salzberg 2012), and base-wise coverage was retrieved with SAMtools (Danecek et al. 2021) and computed with dplyr.

### RNA Isolation and Next-Generation Sequencing

RNA was extracted using an ExtractRNA kit (Evrogen) according to the manufacturer's recommendations. RNA libraries were constructed using the NEBNext Ultra RNA Library Prep Kit by NEB and the NEBNext Multiplex Oligos for Illumina (Dual Index Primers Set 1) by NEB following the manufacturer's protocol. The samples were amplified using 15 PCR cycles. The constructed libraries were sequenced on an Illumina NovaSeq 6000 with a paired-end read length of 61. The obtained 555.3 million reads were trimmed using trimmomatic (Bolger et al. 2014) and mapped to the assembled *P. cf. ciliata* genome using bowtie2, version 2.4.4 (Langmead and Salzberg 2012). The mapped 672,141 reads enabled us to calculate cDNA read depth using SAMtools (Li et al. 2009).

### Phylogenetic Analysis

To analyze the phylogeny of the studied Spionid mitogenomes, we utilized seven mitogenomes available on GenBank as of February 2023, including *P. cf. ciliata*, along with the mitogenome of *Magelona mirabilis* (NC_028711.1) as an outgroup. A total of 9 mt genomes was therefore considered for the phylogenies. We first conducted codon alignments for 12 standard mitochondrial PCGs from the listed mitogenomes. The *ATP8* gene was excluded from the analysis because of its high variability and short length. The alignments were performed using an MSA algorithm integrated into the MACSE tool version 0.9b1 (Ranwez et al. 2011). We then concatenated multiple sequence alignments into a single alignment and used it to build a maximum likelihood (ML) phylogenetic tree using IQ-TREE version 2.2.0.3 (Nguyen et al. 2015).

We partitioned the multiple sequence alignment into 12 segments, each corresponding to a specific gene, and employed partition testing to select the best fit substitution model for each segment. We incorporated rate heterogeneity through the +I+G model. The best fit model was automatically selected by “ModelFinder” based on the Bayesian information criterion (BIC) score. For constructing the consensus tree, we adopted a diverse evolutionary rate strategy. The bootstrap values were determined by 500 replicates, using standard nonparametric bootstrap. The tree was visualized using iTOL6 (https://itol.embl.de/) (fig. 2).

The pipeline that contains several Python scripts for codon gene alignments, alignment concatenation, phylogenetic tree inference, and tree visualization is available in the GitHub repository: https://github.com/MarySelifanova/Mitochondrial-ORFans. The repository also contains gene alignments and other phylogenetics analysis data (data/HKY + I + G_TEST + LMSS_p.zip) as well as more detailed information on phylogenetic inference.

In order to clarify the phylogenetic position of *Polydora* collected in Biofiltry Bay, we also constructed phylogenetic trees of the 16S, 18S, and 28S genes of different *Polydora* species available on GeneBank (supplementary figs. S2–S4, Supplementary Material online). All trees were constructed based on nucleotide alignments using the ML algorithm integrated in MEGA11 software with default parameters, 100 replicates were used to calculate bootstrap values, and the general time reversible model was used as a substitution model.

### Sequence Analysis

Nucleotide and a.a. alignments of standard PCGs and ORFans were performed using the MUSCLE algorithm with default parameters (Edgar 2004) integrated in MEGA11 software (Tamura et al. 2021). Tracks of position conservation were obtained with Jalview 2.11.2 (Waterhouse et al. 2009). Nucleotide and a.a. p-distances, as well as dN/dS ratios and the codon-based test of purifying selection using the Nei-Gojobori method (Nei and Gojobori 1986), were calculated using MEGA11 (Tamura et al. 2021). Sequence comparison plots were built with the ggplot (Wickham 2016) package in R. The codon adaptation index for PCGs was calculated using the Python CAI module (Python Implementation of Codon Adaptation Index) (Lee 2018) with all PCGs of the considered mitogenome set as background. Scripts for the corresponding plots and calculations can be found in the GitHub repository: https://github.com/MarySelifanova/Mitochondrial-ORFans/tree/main.

### Functional Analyses of ORFans

For ORFans, *COX1* and *NAD6* gene domain architectures were obtained using InterProScan 5.59–91.0 (Jones et al. 2014). Hydropathy profiles of a.a. sequences were calculated with the ProtScale tool at ExPASy (Gasteiger et al. 2005), by the method of Kyte and Doolittle (Kyte and Doolittle 1982).

In order to predict possible origins and functions of three ORFans from the *P. cf. ciliata* mitogenome, we performed sequence searches using several search algorithms with default parameters. First, we performed BlastN, TBlastN, and TBlastX searches against NCBI databases. Then, we searched for ORFan homologs using BlastP and PSI-BLAST (Altschul et al. 1997) against either the entire NRPD or UniProtKB (Release 2022_04).

TBlastX and BlastN found no hits in NCBI databases, whereas a TBlastN search found hits in *P. hoplura* mitochondria (e-value: 6e^−28^) and *B. hamata* (e-value: 3e^−17^) mitochondria that are already covered in this study. A BlastP search for ORFans in the *P. cf. ciliata* mitogenome against both NRPD and UniProtKB found no significant hits with e-value cutoff = 1. A PSI-BLAST search against UniProtKB found some bacteria tRNA U34 carboxymethyltransferase hits for one of the ORFans with an e-value no less than 0.5, which we considered insignificant, and the PSI-BLAST search against NRPD resulted in zero hits found.

As suggested in Mitchell et al. (2016), in order to detect more remote possible homologies, we used methods based on profile HMMs. We used hmmsearch 3.3.2 (Potter et al. 2018) to perform a profile HMM–protein sequence comparison with default parameters against Reference Proteomes, UniProtKB, SwissProt, Protein Databank (PDB), and AlphaFold databases, where ORFans’ alignments were used as an input. We also performed a profile HMM–profile HMM comparison against default databases representing proteins with known structure via the latest (as of Dec 2022) version of HHpred with default parameters (Zimmermann et al. 2018).

The tertiary structure prediction, along with the search for structural similarity, was performed using @TOME v3 (Pons and Labesse 2009) and I-TASSER 5.1 (Zheng et al. 2021). In order to address possible functions of ORFan proteins, we searched for known motifs that occur in sequences using MotifScan against default databases (https://myhits.sib.swiss/cgi-bin/motif_scan). Sequences were also searched for tetratricopeptide repeats (TPRs) and pentatricopeptide repeats (PPRs) using TPRpred 11.0 (https://toolkit.tuebingen.mpg.de/tools/tprpred). We checked for possible presence of signal peptides in ORFan proteins using SignalP 3.0 (Bendtsen et al. 2004).

## Supplementary Material

evad219_Supplementary_Data

## Data Availability

*Polydora cf. ciliata* mitogenome is submitted to GenBank, accession number: OQ078742. RNAseq of *P. cf. ciliata* is available in Sequence Read Archive (SRA), accession number SRR26830859. Scripts, intermediate analysis files, and alignments files used in this study are available in the GitHub page: https://github.com/MarySelifanova/Mitochondrial-ORFans/.
