# Supplementary material for: ORFans in Mitochondrial Genomes of Marine Polychaete Polydora
Source: Genome Biol Evol. 2023 Nov 29;15(12):evad219. doi: 10.1093/gbe/evad219 (PMC10721130; doi:10.1093/gbe/evad219)
Supplement: evad219_Supplementary_Data [file evad219_supplementary_data.pdf]

Supplementary information: ORFans in mitochondrial genomes of marine polychaete *Polydora*

Maria Selifanova[1], Oleg Demianchenko[1], Elizaveta Noskova[1], Egor Pitikov[1], Denis Skvortsov[1], Jana Drozd[1], Nika Vatolkina[1], Polina Apel[1], Ekaterina Kolodyazhnaya[1], Margarita A. Ezhova[2,3], Alexander B. Tzetlin[2], Tatiana V. Neretina[1,2,4], Dmitry A. Knorre[2,5]

[1]Faculty of Bioengineering and Bioinformatics, Lomonosov Moscow State University, Russia

[2]N.A. Pertsov White Sea Biological Station, Lomonosov Moscow State University, Russia

[3]Skolkovo Institute of Science and Technology, Moscow, Russia

[4]Institute for Information Transmission Problems of the Russian Academy of Sciences (Kharkevich Institute), Moscow, Russia.

[5]Belozersky Institute of Physico-Chemical Biology, Lomonosov Moscow State University, Russia

Table S1. Long range PCR primers were utilised to selectively amplify mitochondrial DNA fragments. PCR products were sequenced by Illumina. Obtained sequencing reads were then mapped to the mitochondrial genome assembly of *Polydora cf. ciliata* to validate its integrity.

Name	Primer	Tm
F1	TTTTGAACGCCATGAGGGGG	60
R1	GGTTCATCCAGTCCCTGCTC	60
F2	TACTCTCTCCCTCTTTCGCCA	59
R2	AGGTGCAAGCTAGATGTTCTATTT	59
F3	CATCTGGAGCTCCGTCAACA	60
R3	CCTGCTCACCCTCATTGTGT	60
F4	CGACGCAGGACTTCCTTGTT	60
R4	ATCTTCAGTGTGGCGCTCTT	60

Table S2. Primers flanking unannotated regions were utilised to validate the sequences of ORFans and the Control Region (CR) using Sanger sequencing technology.

Name	Primer	Tm	Region
F1	TCCTCAACAGICICCAAICCA	57	1245-1268
R1	ATAGGAGCTGAAGGGGACAT	57	1991-1972
F2	CGCCCAGGTACAGTCTTTGT	59	8716-8735
R2	CTCAATGTTGGGGCATGAC	59	9474-9455
F3	CCTTTGACATCCGAAGTATAG GTA	58	11648-11671
R3	ATAAGCGTTTACCCGAGCAC	58	12906-12887
F4	TTCCCTATCCCCTTAGCACT	58	13856-13857
R4	GCAAGGCCTAGGAGAGACC	58	14799-14780

Table S3. Top 10 @tome v3 (Pons & Labesse 2009) and I-tasser 5.1 (Zheng et al. 2021) hits of the ORFans from *Polydora* and *Boccardiella* species. For @tome v3 results an additional score threshold of 40.00 is also applied, for I-tasser duplicate hits for one protein are excluded. Sequences of the metrics for @tome and I-tasser results are listed in the subsequent headers.

ORFan-1060	ORFan-427	ORFan-544
Atome3 (PDB id, Classification, score, identity)		
P. cf. ciliata<br>4LXU, transferase, 86.04, 18%<br>3ZPJ, unknown function, 71.06, 15<br>1GZ6, dehydrogenase, 67.09, 11%<br>6EU6, membrane protein, 43.64, 30%<br>2OKQ, unknown function, 42.09, 14%,<br>7E2I, lipid transport, 40.73, 30%<br>7E2G, lipid transport, 40.59, 30%<br><br>***P. websteri***<br>3IBT, oxidoreductase, 72.46, 18%<br>3OQ4, cell cycle, 45.68, 9%<br>4TT0, hydrolase, 44.59, 10%<br>2AHQ, transcription, 40.19, 16%,<br>7M8W, membrane protein, 39.94, 33%<br><br>***P. brevipalpa***	***P. cf. ciliata***<br>*2KCD,* unknown function, 77.50, 21%<br>2MN4, de novo protein, 66.92, 20%<br>5JK2, cell adhesion, 64.44, 16%<br>3E0Z, unknown function, 61.57, 20%<br>4DQJ, hydrolase, 60.02, 13%<br>3A98, signaling protein, 59.51, 16%<br>2WCR, immune system, 58.11, 16%<br>4P02, transferase, 50.63, 26%<br>2JKG, protein binding, 49.48, 12%<br>6V93, DNA binding protein, 49.42, 25%<br><br>***P. websteri***<br>3FRR, protein binding, 72.44, 11%<br>4NQI, signaling protein, 64.86, 185<br>2EE3, signaling protein, 64.60, 15%<br>4P02, transferase, 61.76, 31%<br>2I9S, chaperone, 59.82, 17%	***P. cf. ciliata***<br>*4LEU,* RNA binding protein, 78.89, 13%<br>5JJO, immune system, 78.23, 19%<br>4S2R, hydrolase, 70.71, 24%<br>3N0K, hydrolase inhibitor, 70.52, 14%<br>2B6C, unknown function, 66.91, 12%<br>2C0N, viral protein/transferase, 65.40, 17%<br>2VLI, transferase, 62.85, 10%<br>4WIA, ATP-binding protein, 62.43, 11%<br>2QEC, transferase, 61.64, 11%<br>1CA4, TNF signaling, 58.32, 11%<br><br>***P. websteri***<br>*1WCK,* signaling protein,, 70.75, 15%<br>5UGW, transferase, 59.83, 15%

5LRT, hydrolase, 84.97, 18%<br>5I3E, hydrolase, 68.57, 16%<br>5FGU, Metal binding, DNA binding protein, 59.54, 17%<br>3FWK, transferase, 48.62, 13%<br><br>***P. hoplura***<br>5GAP, transcription, 75.96, 17%<br>4RDQ, transport protein, 73.68, 16%<br>4KYI, protein binding/transport protein, 68.26, 15%<br>1PV6, transport protein, 62.45, 9%<br>5FT3, transferase, 53.58, 15%<br><br>***B. hamata***<br>*5T9J,* hydrolase, 77.36, 16%<br>3ALX, viral protein/membrane protein, 75.83, 23%<br>5AHR, hydrolase, 71.38, 22%<br>4BWZ, transport protein, 65.06, 14%<br>5IOJ, hydrolase, 62.28, 16%<br>4IIK, hydrolase, 61.81, 13%<br>3GIA, transport protein, 59.87, 10%<br>5CA8, hydrolase, 59.73, 16%<br>4L4W, protein transport, 58.55, 17%<br>5TCQ, membrane protein, 56.29, 18%	5EJ1, metal binding protein, 58.13, 30%<br>5IFG, hydrolase/antitoxin, 57.77, 13%<br>5E6G, de novo protein, 56.29, 11%<br>6UEB, viral protein, score 53.10, 25%<br>2HEQ, unknown function, 50.24, 19%<br><br>***P. brevipalpa***<br>*3A98,* signaling protein, 71.18, 20%<br>2MN4, de novo protein, 60.39, 22%<br>7BJK, oxidoreductase, 59.34, 27%<br>2DDZ, unknown function, 51.35, 11%<br>2FM9, cell invasion, 49.77, 11%<br>4P02, transferase, 43.47, 29%<br>5EJ1, metal binding protein, 42.24, 29%<br>1VI7, unknown function, 41.78, 15%<br>4P1Z, RNA binding protein, 40.57, 10%<br><br>***P. hoplura***<br>*5M11,* transport protein, 66.24, 21%<br>3A98, signaling protein, 61.49, 14%<br>4R6I, transcription, 60.98, 19%<br>4ZOX, chaperone, 60.33, 13%<br>5IFG, hydrolase/antitoxin, 57.83, 17%<br>2EE3, signaling protein, 55.40, 17%<br>2KCD, unknown function, 51.55, 18%<br>1JHU, transferase, 50.97, 6%<br>6WLZ, membrane protein, 40.35, 42%<br><br>***B. hamata***<br>1UG7, structural genomics/unknown function, 86.09, 19%<br>6BWI, membrane protein, 72.75, 30%<br>6BQV, membrane protein, 62.20, 30%<br>6BCO, transport protein, 62.03, 30%<br>6BQR, 61.23, 30%<br>5WP6, membrane protein, 59.71, 30%<br>2EDB, apoptosis, 58.28, 20%<br>1BCF, iron storage and electron transport, 57.79, 9%<br>3WX4, viral protein, 56.92, 12%<br>2ND4, hydrolase receptor, 54.61, 11%	2VLI, transferase, 56.06, 9%<br>1ZWT, cell adhesion, 50.49, 20%<br>1J3G, hydrolase, 49.91, 13%<br>2EDO, cell adhesion, 49.75, 14%<br>2E33, lactase/hydrolase, 49.48, 25%<br>1UMI, ligase, 48.42, 25%<br>2E31, ligase, 48.12, 25%<br>1UMH, ligase, 47.95, 25%<br>2RJ2, ligase, 47.58, 25%<br><br>***P. brevipalpa***<br>2OH5, structure protein/RNA binding protein, 68.10, 18%<br>3G8Q, RNA binding protein, 67.52, 18%<br>2AN1, transferase, 61.52, 11%<br>5LQ6, immunosuppressant, 58.64, 14%<br>3N0K, hydrolase inhibitor, hydrolase, 14%<br>2WWX, protein transport, 54.03, 18%<br>4G6V, toxin, 53.12, 15%<br>4NQI, signaling protein, 52.97, 12%<br>3HG9, unknown function, 50.09, 17%<br>4FPR, protein binding, 49.00, 20%<br><br>***P. hoplura***<br>3G8Q, RNA binding protein, 71.71, 16%<br>3E9C, hydrolase, 66.33, 15%<br>3DCM, transferase, 64.91, 14%<br>3WG9, transcription, 61.86, 16%<br>5ANB, translation, 57.21, 18%<br>2AMJ, oxidoreductase, 54.93, 10%<br>2FO1, gene regulation/signaling protein, 54.28, 18%<br>1WBL, lectin, 52.85, 15%<br>2C0N, viral protein/transferase, 47.76, 19%<br>2OST, hydrolase, 46.40, 15%<br><br>***B. hamata***<br>4QGN, oxidoreductase, 73.33, 13%<br>5A9H, transport protein, 72.68, 13%<br>1CA4, TNF signaling, 71.32, 11%<br>5FT0, hydrolase inhibitor, 68.88, 18%<br>5B5Z, metal binding protein, 65.53, 14%<br>3KEA,viral protein, 64.49, 19%<br>5ARM, copper-binding protein, 63.63, 13%<br>4PHR, transferase, 61.79, 18%
I-tasser (PDB id, Classification, identity1, identity2, coverage)		
P. cf. ciliata<br>3H1I, oxidoreductase, 0.18, 0.18, 0.82<br>2PFF, transferase, 0.40, 0.27, 0.06<br>6FVB, nuclear protein, 0.16, 0.30, 0.78<br>7DBG, transport protein, 0.14, 0.19, 0.92<br>3ZKV, transport protein, 0.12, 0.28, 0.86<br>7MEX, transferase, 0.13, 0.27, 0.95	***P. cf. ciliata***<br>4FGV, transport protein, 0.15, 0.16, 0.75<br>7WKK, structural protein, 0.11, 0.50, 0.99<br>1HS6, hydrolase, 0.18, 0.19, 0.92<br>3JAC, metal transport, 0.18, 0.31, 0.41<br>5H3O, transport protein, 0.13, 0.22, 0.95	***P. cf. ciliata***<br>4N5Q, protein binding, 0.11, 0.18, 0.72<br>7SZX, viral protein, 0.10, 0.17, 0.85<br>6Q8J, splicing, 0.14, 0.19, 0.90<br>4V4L, apoptosis, 0.37, 0.38, 0.15<br>4ZGN, cell cycle, 0.17, 0.17, 0.72<br>4DR0, oxidoreductase, 0.18, 0.24, 1.0

2XWU, ligase/nuclear protein, 0.13, 0.28, 0.91
7WSS, hydrolase, 0.13, 0.21, 0.97
5NVR, structural protein, 0.16, 0.29, 0.93
7QJ0, cytosolic protein, 0.12, 0.22, 0.94

P. websteri
4RY2, transport protein/hydrolase, 0.14, 0.20, 0.83
2PFF, transferase, 0.29, 0.28, 0.81
6FVB, nuclear protein, 0.13, 0.30, 0.96
7DBG, transport protein, 0.12, 0.20, 0.91
3QF4, transport protein, 0.12, 0.17, 0.81
3JAC, metal transport, 0.19, 0.26, 0.91
6N1Z, transport protein, 0.11, 0.26, 0.86

P. brevipalpa
4RY2, transport protein/hydrolase, 0.10, 0.22, 0.82
5T8V, cell cycle, 0.15, 0.23, 0.88
7DBG, transport protein, 0.16, 0.19, 0.89
6KG7, membrane protein, 0.16, 0.17, 0.88
5WYL, ribosomal protein/nuclear protein, 0.16, 0.20, 0.71
7WKK, structural protein, 0.15, 0.22, 0.84
5DLQ, protein transport, 0.16, 0.30, 0.88
7OCI, membrane protein, 0.15, 0.25, 0.96
5U1T, hydrolase, 0.18, 0.32, 0.79
7QE7, cell cycle, 0.17, 0.21, 0.94

P. hoplura
6WW2, membrane protein, 0.12, 0.24, 0.88
2PFF, transferase, 0.27, 0.25, 0.13
5UFL, signaling protein, 0.17, 0.25, 0.88
7DBG, transport protein, 0.14, 0.18, 0.92
5NL2, membrane protein, 0.14, 0.20, 0.86
6FVB, nuclear protein, 0.13, 0.35, 0.91
7WKK, structural protein, 0.15, 0.23, 0.95
7P5C, lipid transport, 0.10, 0.21, 0.81
5OQQ, cell cycle, 0.18, 0.27, 0.80

B. hamata
3JAC, metal transport, 0.21, 0.25, 0.88
2XWU, ligase/nuclear protein, 0.14, 0.29, 0.58
7OCI, membrane protein, 0.14, 0.27, 0.99
4V4L, apoptosis, 0.24, 0.12, 0.42
4HAT, protein transport/antibiotic, 0.22, 0.33, 0.57
7W7G, membrane protein, 0.18, 0.23, 0.92
1W27, lyase, 0.15, 0.27, 0.85
7ESI, hydrolase, 0.14, 0.21, 0.95

5NL2, membrane protein, 0.11, 0.17, 0.98
4V4L, apoptosis, 0.25, 0.35, 0.98
5UFK, protein binding, 0.15, 0.25, 0.86
7ZCV, DNA binding protein, 0.12, 0.30, 0.99

P. websteri
6W6X, de novo protein, 0.14, 0.17, 0.77
7M68, membrane protein, 0.09, 0.42, 0.98
2MN2, antitoxin, 0.18, 0.18, 0.87
4HG6, transferase, 0.32, 0.23, 0.44
7LP9, membrane protein, 0.08, 0.17, 0.96
5NL2, membrane protein, 0.08, 0.22, 0.1
5DN6, hydrolase, 0.15, 0.12, 0.45
5WZJ, RNA binding protein/RNA, 0.14, 0.30, 0.96
7ZCV, DNA binding protein, 0.15, 0.25, 0.96

P. brevipalpa
4MU6, unknown function, 0.16, 0.19, 0.88
7W7G, membrane protein, 0.09, 0.24, 0.90
4ZQB, oxidoreductase, 0.16, 0.26, 0.90
5MZ4, viral protein, 0.53, 0.26, 0.12
6O02, transferase/protein binding 0.25, 0.18, 0.86
3JBR, membrane protein, 0.08, 0.13, 1.0
2PFF, transferase, 0.25, 0.32, 0.91
5UFK, protein binding, 0.21, 0.28, 0.86
7ZCV, DNA binding protein, 0.15, 0.18, 0.94

P. hoplura
1ZKR, allergen, 0.14, 0.17, 0.82
7M68, membrane protein, 0.12, 0.42, 1.0
4EJT, transcription regulator/RNA
2AFF, cell cycle, 0.53, 0.08, 0.11
4XMN, protein transport, 0.17, 0.19, 0.91
6XR1, de novo protein, 0.07, 0.19. 1.0
3JAC, metal transport, 0.15, 0.30, 0.98
6R7O, gene regulation, 0.17, 0.26, 0.96
7VOI, hydrolase, 0.19, 0.26, 0.95

B. hamata
1T33, transcription, 0.13, 0.15, 0.79
7N0A, cytokine/immune system, 0.14, 0.19, 0.99
6TV5, structural protein, 0.15, 0.18, 0.94

6TGB, signaling protein, 0.17, 0.12, 0.50

2MX8, structural protein, 0.09, 0.17, 0.90
6BQ1, transferase/signaling protein, 0.20, 0.42, 0.98
6IGX, cell cycle, 0.13, 0.38, 1.0
7U7N, cytokine, 0.15, 0.16, 0.96

3JAC, metal transport, 0.18, 0.27, 0.93
6DJY, virus, 0.11, 0.37, 0.78
7VOI, hydrolase, 0.22, 0.25, 0.89

P. websteri
2J5T, transferase, 0.14, 0.16, 0.88
7NQD, transferase, 0.06, 0.19, 0.89
4C47, cell adhesion, 0.20, 0.19, 0.94
1D3Y, isomerase, 0.19, 0.14, 0.26
6LF6, transferase, 0.24, 0.17, 0.68
6ZP9, viral protein, 0.09, 0.17, 0.96
4V4L, apoptosis, 0.27, 0.16, 0.76
5UN8, hydrolase, 0.18, 0.27, 0.85
7P6G, hydrolase, 0.15, 0.23, 0.92

P. brevipalpa
5YO8, lyase, 0.15, 0.12, 0.*67*
7BZX, transferase, 0.13, 0.28, 0.94
5ZMO, DNA binding protein/DNA, 0.13, 0.20, 0.82
2PFF, transferase, 0.20, 0.36, 0.16
7KB3, signaling protein, 0.20, 0.20, 0.97
6W2V, biosynthetic protein, 0.11, 0.16, 0.98
2R7G, transcription repressor/cell cycle, 0.19, 0.25, 0.70
7VOI, hydrolase, 0.20, 0.23, 0.94

P. hoplura
2PFF, transferase, 0.31, 0.32, 0.85
4V4L, apoptosis, 0.25, 0.18, 0.71
2LF6, signaling protein,0.21, 0.14, 0.43
7FIV, protein binding, 0.12, 0.16, 0.78
1H6U, cell adhesion, 0.17, 0.20, 0.96
3CJH, protein transport, 0.23, 0.07, 0.14
6HXZ, virus like particle, 0.24, 0.17, 0.81
6W2V, biosynthetic protein, 0.07, 0.16, 0.97
3JAC, metal transport, 0.17, 0.33, 0.80

B. hamata
2PFF, transferase, 0.33, 0.31, 0.11
4V4L, apoptosis, 0.24, 0.31, 0.89
1HRK, lyase, 0.16, 0.16, 0.69
7NHA, viral protein, 0.16, 0.21, 0.87
4R7Q, signaling protein, 0.20, 0.22, 0.96

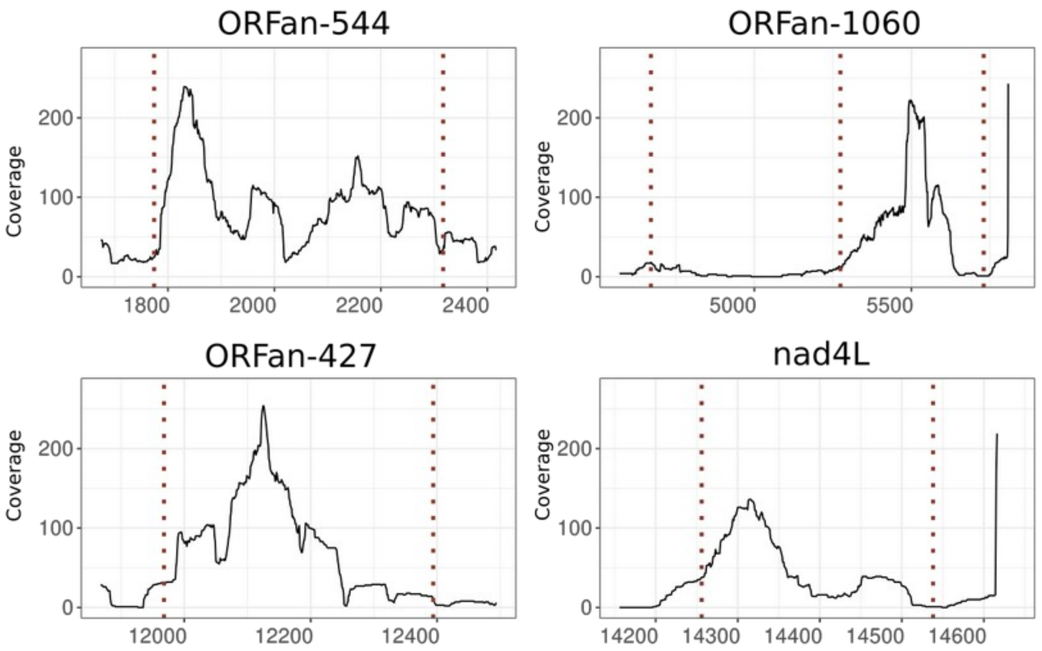

Figure S1. RNAseq read coverage of the ORFans regions. nad4L gene is shown for comparison. Red dotted lines represent gene boundaries. For ORFan-1060 an alternative position is indicated at position 5274 – this transcription start position was predicted by StringTie using RNAseq data.

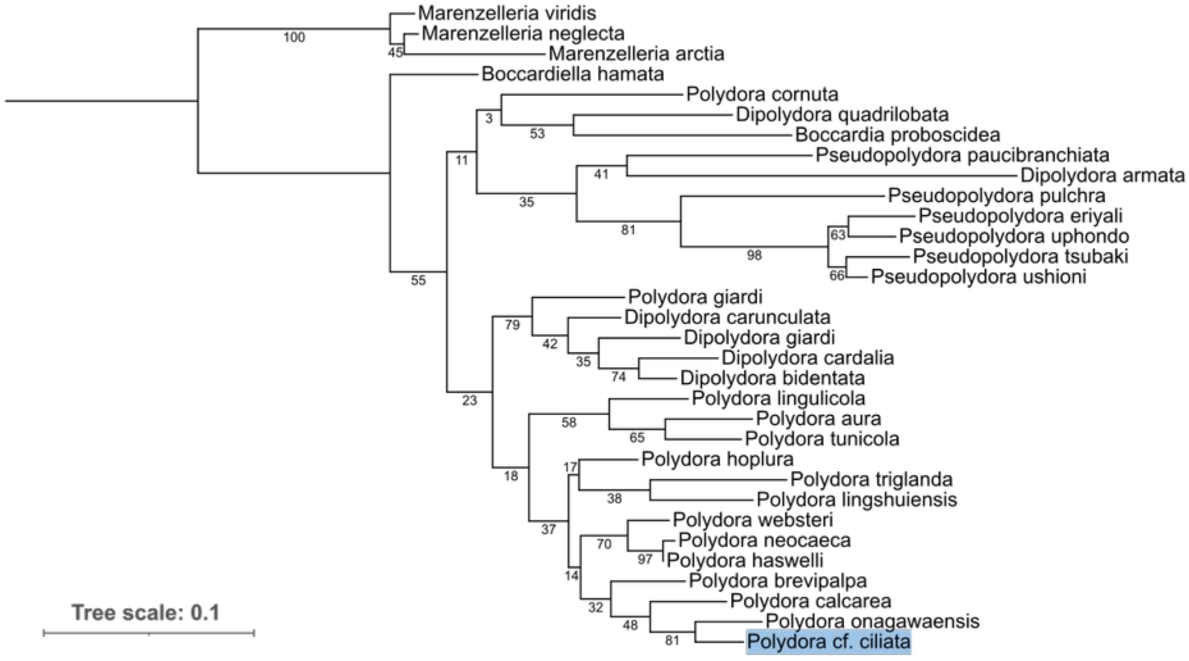

Figure S2. Phylogenetic tree of 16S sequences of *Polydora cf. ciliata* and related Annelida species constructed using the Maximum Likelihood (ML) algorithm integrated in MEGA11 software with General Time Reversible substitution model with default parameters and 100 bootstrap replicas.

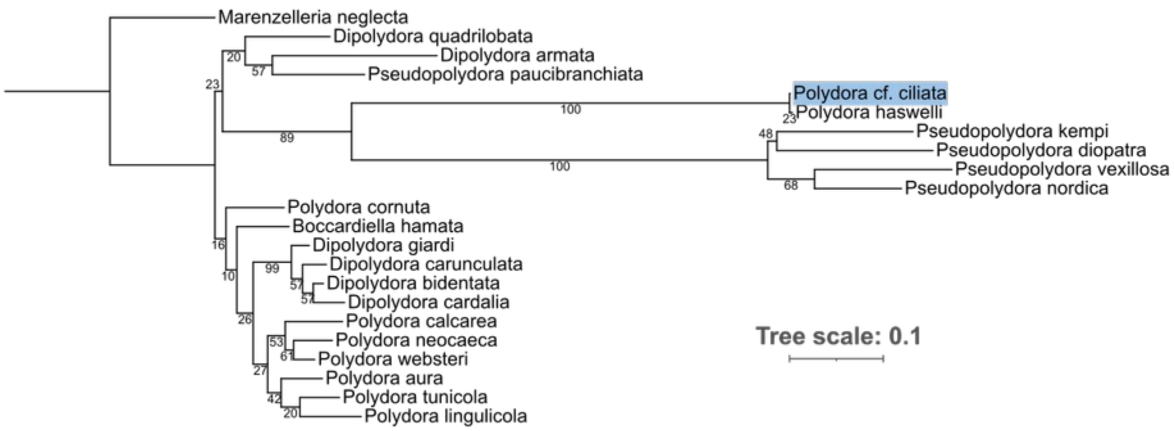

Figure S3. Phylogenetic tree of 18S sequences of *Polydora cf. ciliata* and related Annelida species constructed using the Maximum Likelihood (ML) algorithm integrated in MEGA11 software with General Time Reversible substitution model with default parameters and 100 bootstrap replicas.

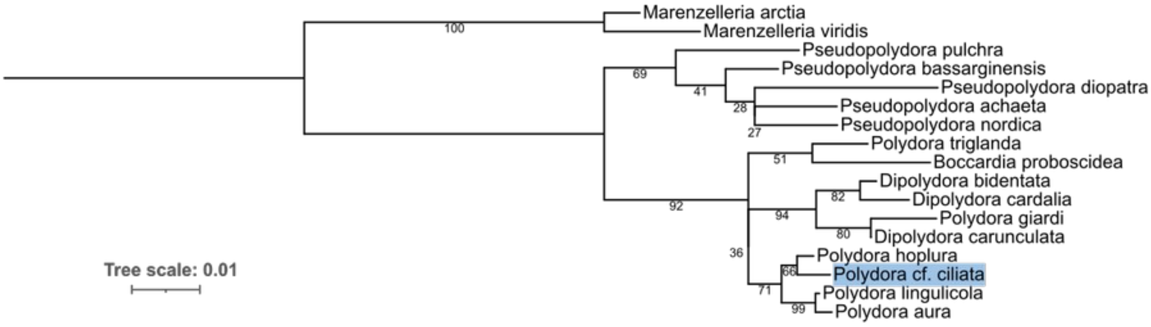

Figure S4. Phylogenetic tree of 28S sequences of *Polydora cf. ciliata* and related Annelida species constructed using the Maximum Likelihood (ML) algorithm integrated in MEGA11 software with General Time Reversible substitution model with default parameters and 100 bootstrap replicas.

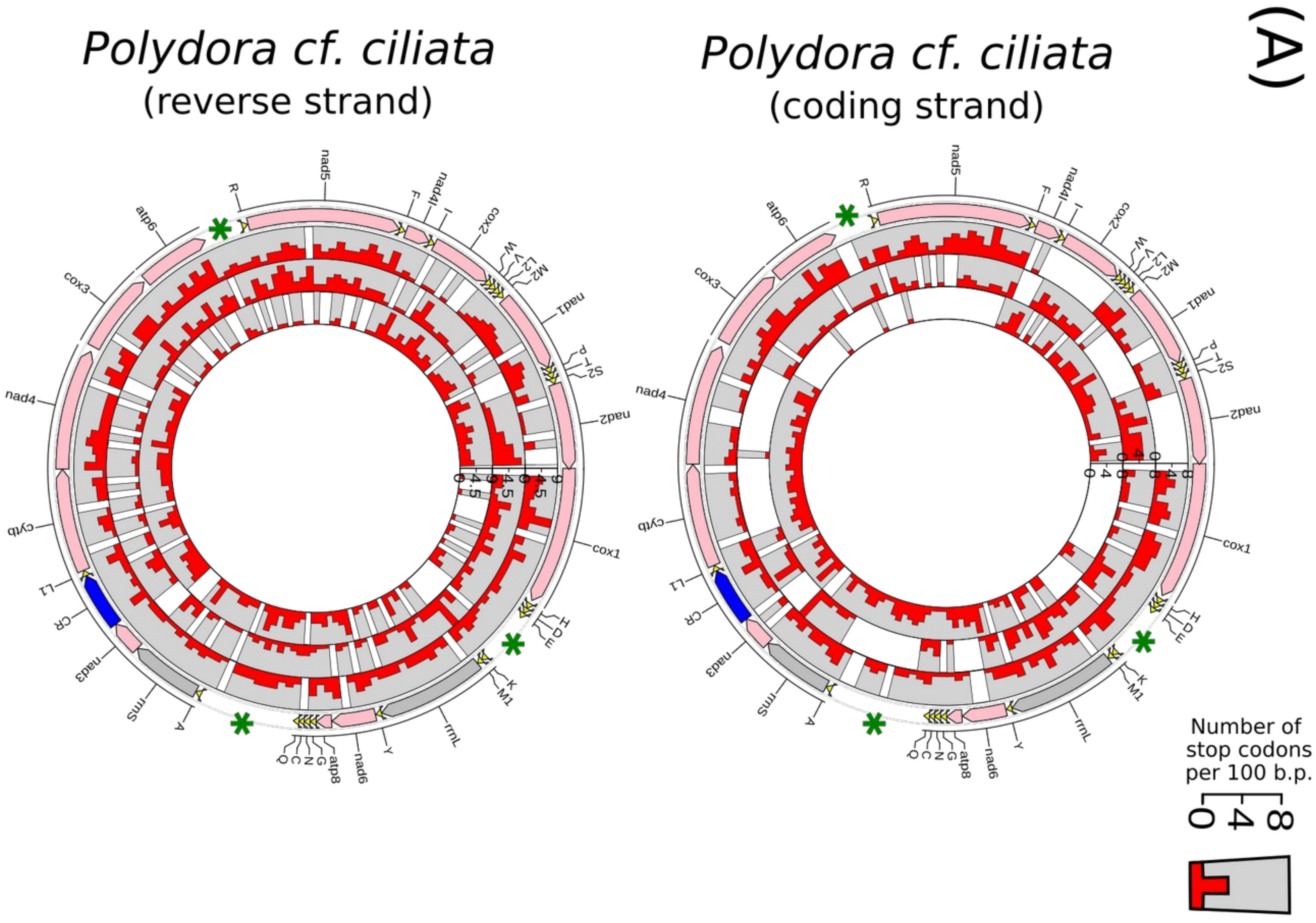
(A)
Polydora cf. ciliata
(reverse strand)
Polydora cf. ciliata
(coding strand)
Number of
stop codons
per 100 b.p.
0
4
8
cox1
nad2
S2
T
P
nad1
M2
L2
V
W
cox2
I
nad4l
F
nad5
R
atp6
cox3
nad4
cytb
L1
CR
nad3
rrnS
A
Q
C
N
G
atp8
nad6
Y
rrnL
M1
K
E
D
H

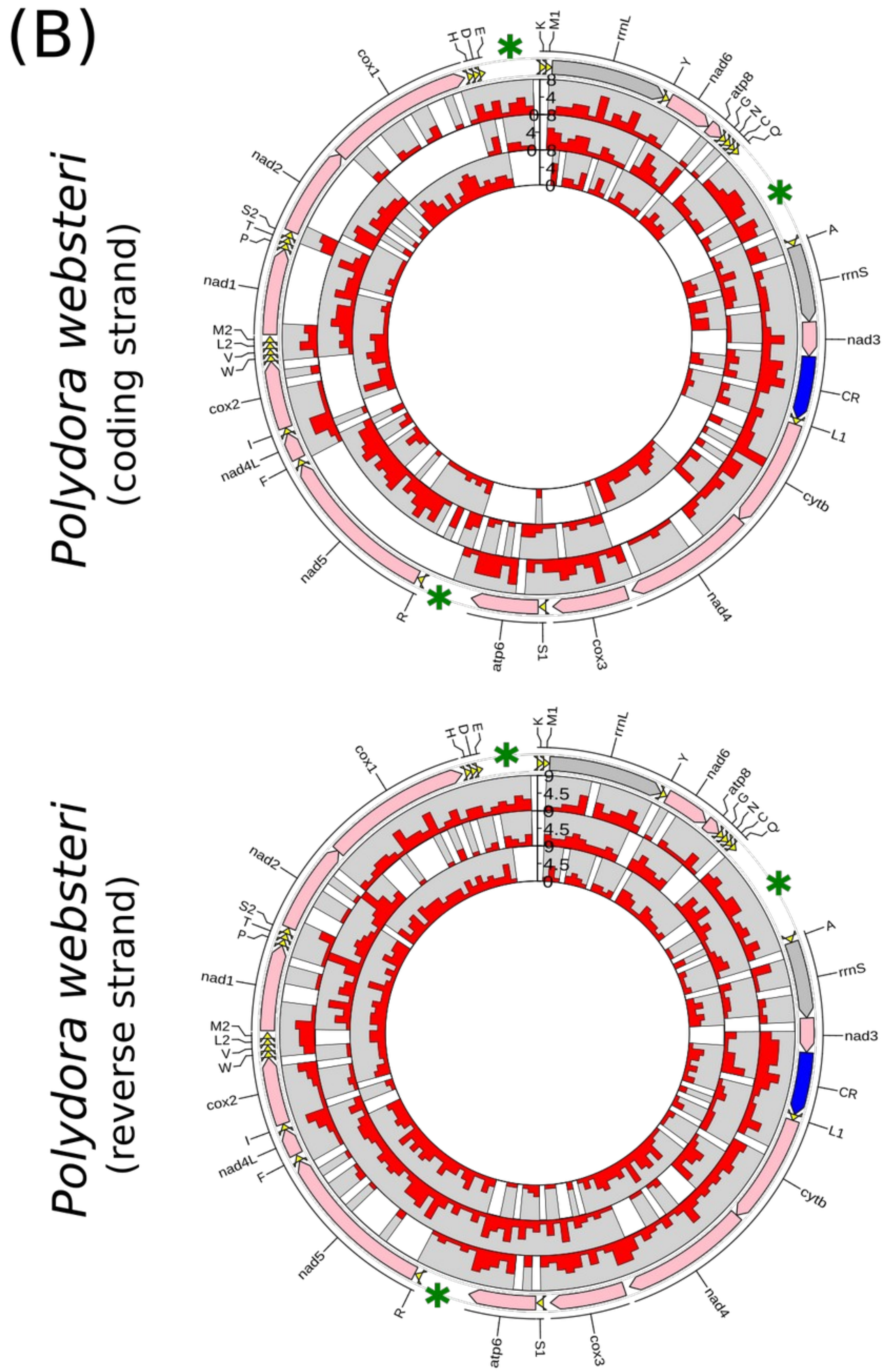

(B)
Polydora websteri
(coding strand)
Polydora websteri
(reverse strand)
K
M1
rrnL
Y
nad6
atp8
G
N
C
Q
A
rrnS
nad3
CR
L1
cytb
nad4
cox3
S1
atp6
R
nad5
F
nad4L
I
cox2
W
V
L2
M2
nad1
P
T
S2
nad2
cox1
H
D
E

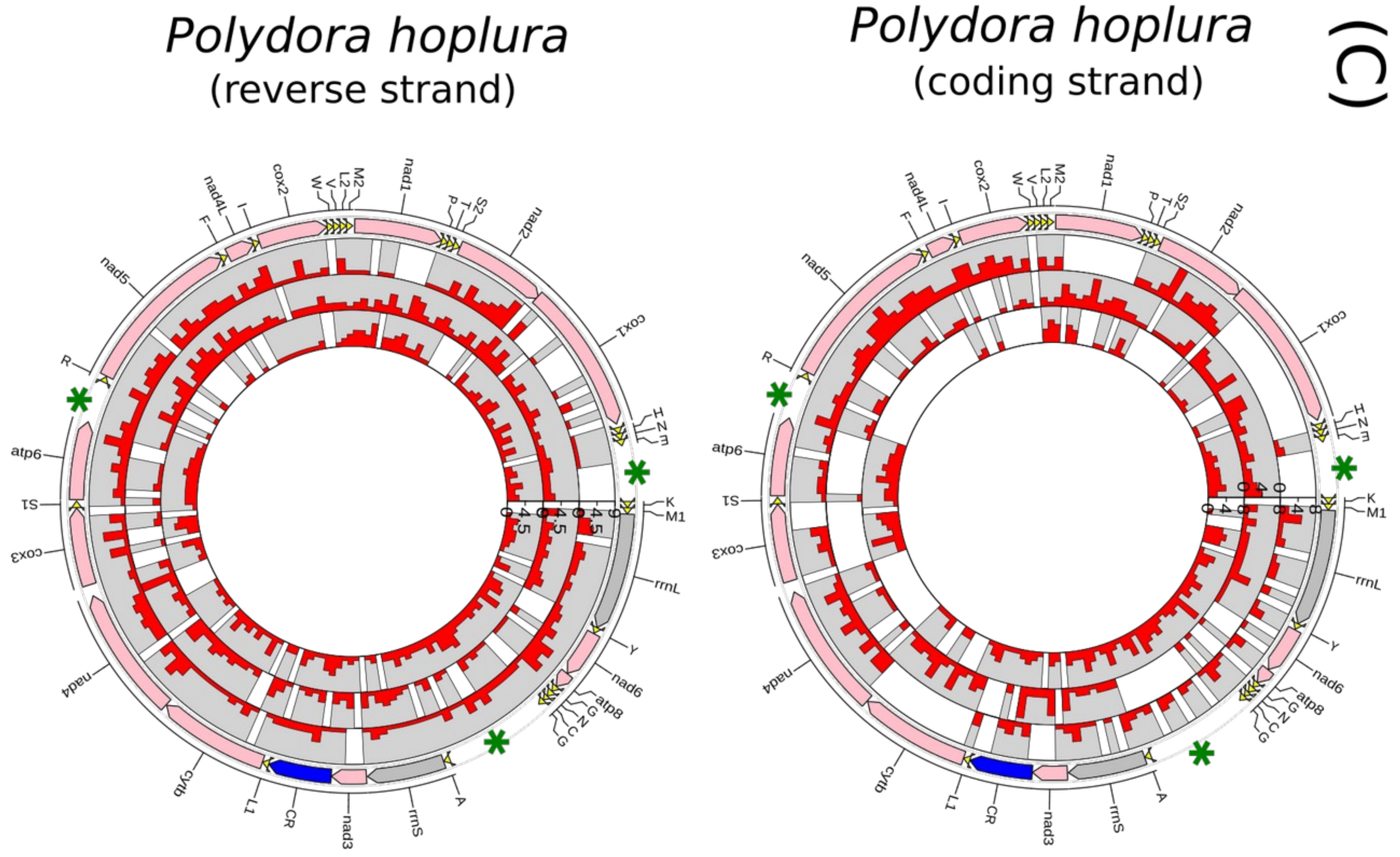
(C)
Polydora hoplura
(reverse strand)
Polydora hoplura
(coding strand)
K
M1
rrnL
Y
nad6
atp8
G
N
C
G
A
rrnS
nad3
CR
L1
cytb
nad4
cox3
S1
atp6
R
nad5
F
nad4L
I
cox2
W
V
L2
M2
nad1
P
T
S2
nad2
cox1
H
N
E
9
4.5
0
8
4
0

(D)

Polydora brevipalpa
(reverse strand)

Polydora brevipalpa
(coding strand)

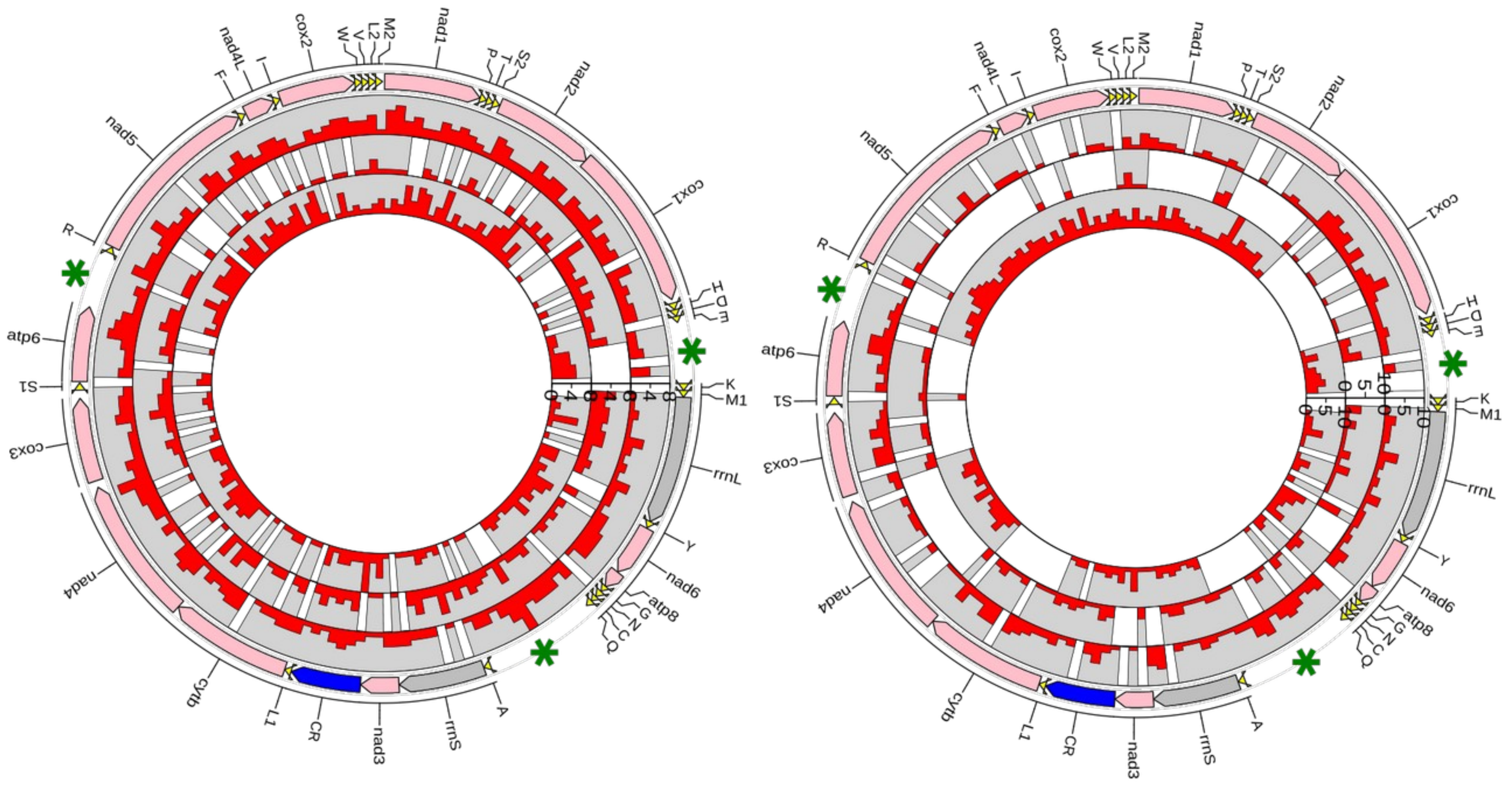

(E)

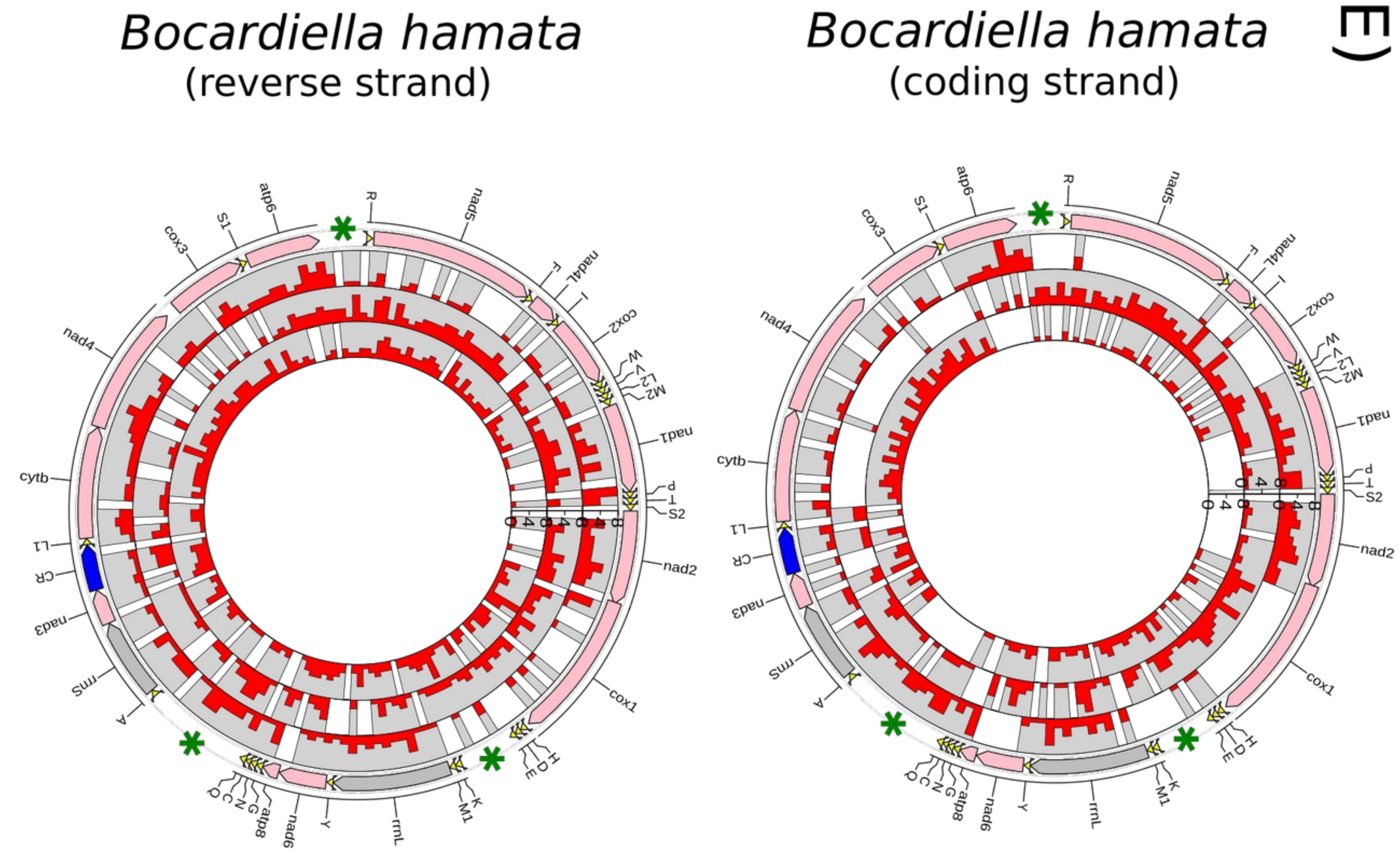
Bocardiella hamata
(reverse strand)
Bocardiella hamata
(coding strand)
nad5
R
atp6
S1
cox3
nad4
cytb
L1
CR
nad3
rrnS
A
Q
C
N
G
atp8
nad6
Y
rrnL
M1
K
E
D
H
cox1
nad2
S2
T
P
nad1
M2
L2
V
W
cox2
I
nad4L
F
8
4
0

Figure S5. Maps of all analysed mitogenomes with visualised stop codons in six reading frames. Outer circle shows annotation for a particular genome; the positions of the ORFans are indicated by the green asterisks. Three mid-circles show the amount of stop codons per 100 b.p. bin for each reading frame in a coding strand (see legend). Note that stop codons can be annotated in the first and last bins of the protein coding genes because a single bin may overlap with both protein-coding sequences in the right frame, and a NCR or coding region in a non-coding frame.

A

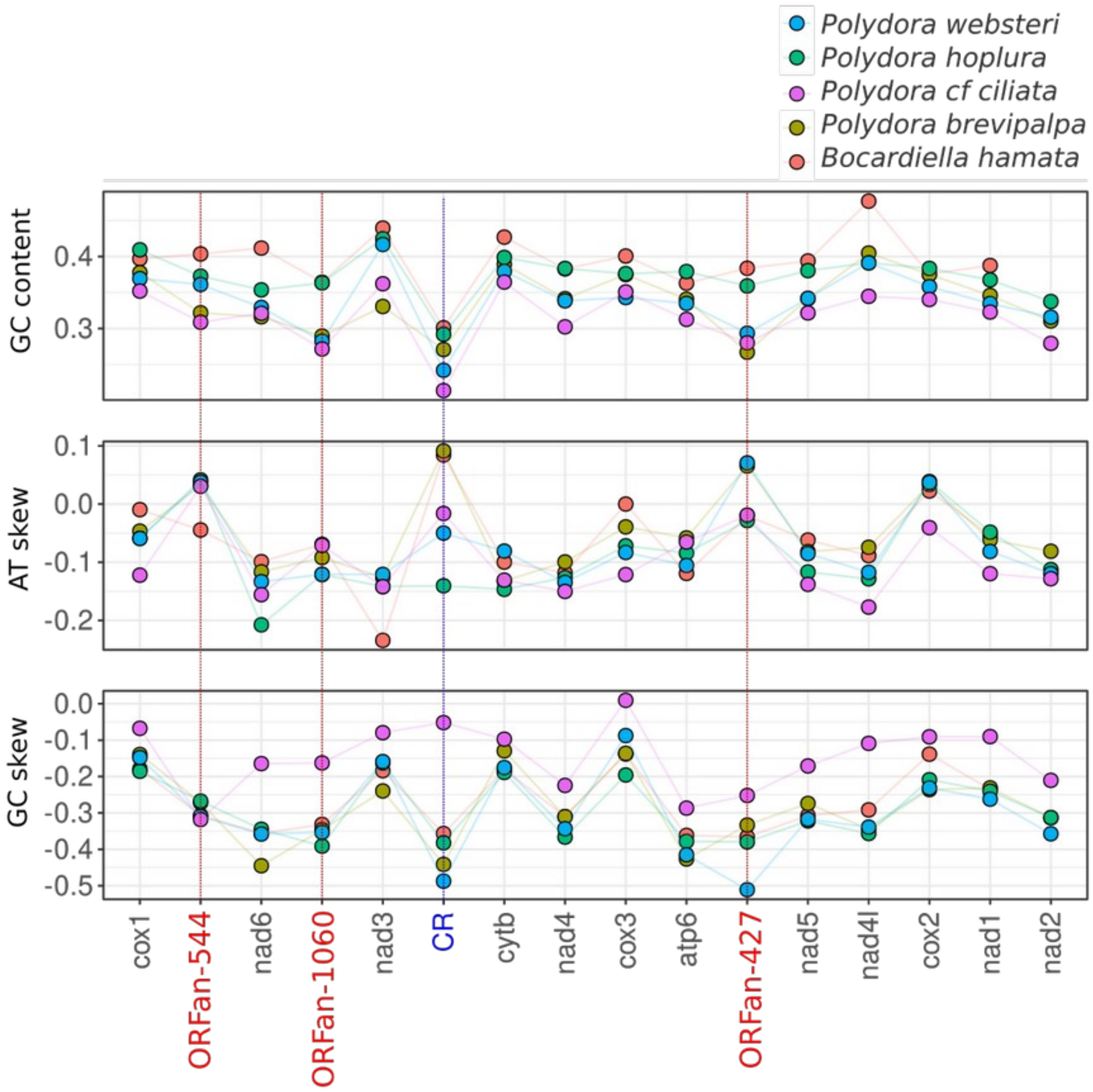
Polydora websteri
Polydora hoplura
Polydora cf ciliata
Polydora brevipalpa
Bocardiella hamata
GC content
AT skew
GC skew
cox1
ORFan-544
nad6
ORFan-1060
nad3
CR
cytb
nad4
cox3
atp6
ORFan-427
nad5
nad4l
cox2
nad1
nad2

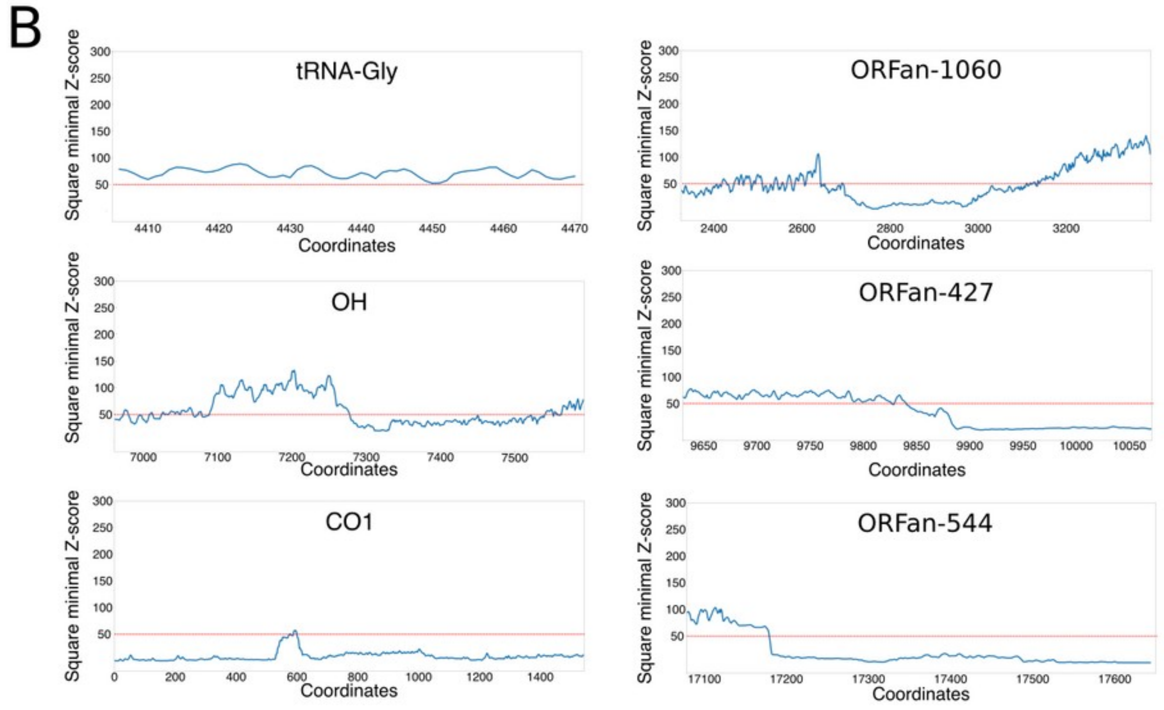

Figure S6. Genomic statistics for individual genes in Polydora and Bocardiella mitochondrial genomes. A. Analysis of the nucleotide content of *Polydora* and Bocardiella mitochondrial genes and putative control region (CR) (GC-content, A-T and G-C skew) **B.** Analysis of the nucleotide sequence for its ability to form secondary structures in a single-stranded state. We analysed three ORFan genes, tRNA-Gly, CO1, and a possible control region. The ability of a sequence to form a stable secondary structure was measured by computing Z-scores; higher values of square minimum Z-score reflect a higher significance of secondary structure in the region. The possible presence of regulatory secondary structures was addressed using RNASurface server (Soldatov, Vinogradova, and Mironov 2014).

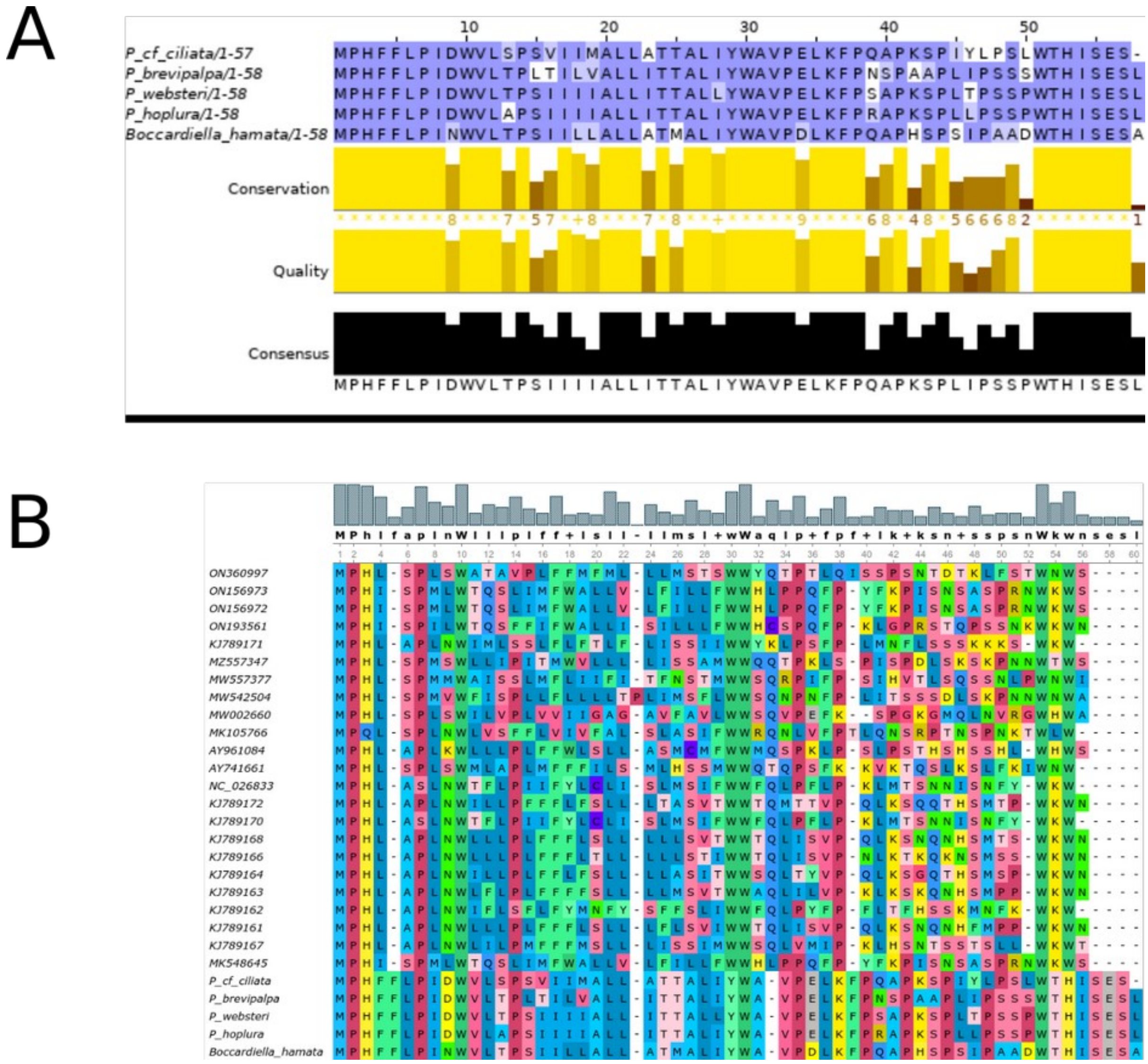

Figure S7. Multiple alignments of ATP8 genes. Given the highly conservative nature of mitogenome architecture in the *Polydora* genus and *Bocardiella hamata*, we searched for atp8 right after nad6 in the same position where the gene was located in species with annotated atp8. We performed multiple alignment of atp8 genes from *Polydora* and *Bocardiella* species [Muscle with defaults parameters, JalView] (A). The sequences proved to be nearly identical, thus we concluded that these ORFs are indeed atp8 sequences not found by automatic annotation. We conducted multiple alignment of all known sedentarian atp8 sequences [using Muscle algorithm with defaults parameters] (B). The alignment revealed substitution of the conserved part of the protein in *Sedentaria*: the first four residues consensus appears to be MPHL instead of MPQL. Although we see the same three first a.a. residues in the *Polydora* genus, its atp8 exhibits exceptional differences from other sedentarian species.

Supplementary text 1. Description of remote homology search algorithms outputs.

In order to detect more remote homologies we performed HMM vs sequence search using hmmsearch against available databases (see methods). As a result of the search, no significant hits (with the cutoff value of 0.01 as suggested by the program) were found in any of the databases for both ORFan-1060 and ORFan-427. For ORFan-544, hmmsearch found one significant hit (e-value=0.0028) in the ‘reference proteomes' database and two significant hits in the UniProtKB (e-values=0.0009 and 0.0083). All hits were “uncharacterised proteins” either from *Tenericutes bacterium* (recommended name: CARDB domain-containing protein) or *Cinara cedri* (recommended name: RNA-directed DNA polymerase).

HMM vs HMM search performed by HHpred revealed several possible homologies, though with a probability not exceeding 70%. As suggested by HHpred, we considered hits with > 50% and hits which are among top three hits with > 30% probability. Given the suggested cutoff, three hits for ORFan-1060 were found: RNA-binding protein 42 from *Homo sapiens*, Putative gene 60 protein from *Bacillus subtilis*, and Uncharacterized protein yaiA from *Escherichia coli*. These hits hardly indicate the function of the protein studied. For ORFan-427, 21 hits were found while the top seven hits with similar probability (approximately 60%) were proteins connected with ABC transporters from different bacteria. Among other hits there were also several transport and membrane proteins. These findings suggest that ORFan2 may be connected with membrane transport. No hits were found for ORFan-544.

Tertiary structure prediction and search for structure similarity for three ORFans in five species was performed using @tome. For ORFan-1060, proteins were connected with the hydrolase family in all species studied. However, the level of similarity does not exceed 40% and the significance score does not exceed 30 in any of the cases. Some hits are connected with transmembrane proteins associated with transport, especially in *Polydora cf. ciliata*. For ORFan--427, transferases or parts of transferases were found in all species. Although these findings were insignificant, these results are consistent with the results of HHpred which pointed on ABC transporters. For ORFan-544,hydrolases, transferases and dna-binding proteins were among hits in all species, however percentage identity and score were substantially low. Interestingly, @tome found a signal peptide in ORFan-544 of *Polydora brevipalpa*, which is consistent with TOPCONs results even though the prediction algorithms are different. More detailed report on @tome results is shown in Table S3.

Taken together, our search for distant homologies across all three ORFans sequences from five different species produced only inconsistent results: the distant homologies found for one ORFan sequence in one species never displayed the homologies with the corresponding ORFan sequence from other *Polydora* and *Bocardiella* species. Therefore, the inconsistencies found in the taxonomy and diverse functions of the proteins, coupled with the relatively weak scores of the hits for ORFans from different species, suggest that the detected putative homologies are false positives.

Supplementary text 2. Author Contributions

All authors participated in conceptualisation, manuscript writing, and approved the final version of the manuscript.

Maria Selifanova — project supervision, formulation of research goals and aims, data review and validation, draft of original manuscript text, preparation of the illustrations
Oleg Demianchenko – genome assembly, annotation plots, identification of ORFans (stop-codon plots), sequence statistics comparison of protein-coding genes, p-distances and dn/ds comparison of protein-coding genes, data review
Elizaveta Noskova – primer design, plots for domain architecture and alignments, reports on general sequence search, clarification of ORFans boundaries
Egor Pitikov – genome assembly and annotation, assembly polishing, phylogenetic trees, CAI calculation, structured segments in sequences
Denis Skvortsov – literature search, automatic and manual genome annotation and submission to GeneBank, atp8 identification, genes' alignments and characterisation of protein-coding genes, plots for genome architecture, RNAseq analysis, verification of tRNAs predictions.
Jana Drozd – domain search, identification of signal peptides, hydropathy profiles of amino acid sequences, some NGS data analysis, functional analysis of ORFans
Nika Vatolkina – distant homology HMM search, structural and functional analysis of ORFans, literature analysis, search for possible viral origin
Polina Apel – primer design, characterisation of protein-coding genes, hydropathy profiles adjustment for alignments
Ekaterina Kolodyazhnaya – evolutionary data analysis
Margarita Ezhova — library preparations and sequencing, verification PCRs and Sanger sequence reactions.
Alexander B. Tzetlin — drafting original manuscript text, specimens identification
Tatiana V. Neretina — project supervision, formulation of research goals and aims, sequencing and library preparations, verification PCRs and Sanger sequence reactions, project administration, funding acquisition
Dmitry A. Knorre — project supervision, formulation of research goals and aims, drafting original manuscript text, preparation of illustrations.
